# Prefrontal-Hippocampal Pathways Through the Nucleus Reuniens Are Functionally Biased by Brain State

**DOI:** 10.3389/fnana.2021.804872

**Published:** 2022-01-31

**Authors:** Brandon E. Hauer, Silvia Pagliardini, Clayton T. Dickson

**Affiliations:** ^1^Neuroscience and Mental Health Institute, University of Alberta, Edmonton, AB, Canada; ^2^Department of Physiology, University of Alberta, Edmonton, AB, Canada; ^3^Department of Anesthesiology and Pain Medicine, University of Alberta, Edmonton, AB, Canada; ^4^Department of Psychology, University of Alberta, Edmonton, AB, Canada

**Keywords:** urethane (carbamate), non-REM, theta, slow oscillation, REM (rapid eye movement), memory–consolidation

## Abstract

Circuit-level communication between disparate brain regions is fundamental for the complexities of the central nervous system operation. Co-ordinated bouts of rhythmic activity between the prefrontal cortex (PFC) and hippocampus (HPC), in particular, are important for mnemonic processes. This is true during awake behavior, as well as during offline states like sleep. We have recently shown that the anatomically interposed thalamic nucleus reuniens (RE) has a role in coordinating slow-wave activity between the PFC and HPC. Here, we took advantage of spontaneous brain state changes occurring during urethane anesthesia in order to assess if PFC-HPC communication was modified during activated (theta) vs. deactivated (slow oscillation: SO) states. These forebrain states are highly similar to those expressed during rapid eye movement (REM) and non-REM stages of natural sleep, respectively. Evoked potentials and excitatory current sinks in the HPC were consistently larger during SO states, regardless of whether PFC or RE afferents were stimulated. Interestingly, PFC stimulation during theta appeared to preferentially use a cortico-cortical pathway, presumably involving the entorhinal cortex as opposed to the more direct RE to HPC conduit. Optogenetic and chemogenetic manipulations of the RE suggested that this state-dependent biasing was mediated by responding in the RE itself. Finally, the phase of both ongoing rhythms also appeared to be an important factor in modulating HPC responses, with maximal field excitatory postsynaptic potentials (EPSPs) occurring during the negative-going phase of both rhythms. Thus, forebrain state plays an important role in how communication takes place across the PFC and HPC, with the RE as a determining factor in how this is shaped. Furthermore, ongoing sleep-like rhythms influence the coordination and perhaps potentiate excitatory processing in this extended episodic memory circuit. Our results have direct implications for activity-dependent processes relevant to sleep-dependent memory consolidation.

## Introduction

Coordinated neural activity is vital for mnemonic processes (Buzsaki, 1996; Siapas and Wilson, 1998). One of the most widely studied phenomena in memory-relevant brain areas is the emergence (and prevalence) of collective oscillatory activity. These rhythmic patterns and their inter-regional synchrony are thought to modulate and constrain information processing, especially in key memory centers including the hippocampus (HPC) and medial prefrontal cortex (mPFC). Decades of research have indicated a decisive role of both HPC and mPFC in episodic mnemonic processes (Jin and Maren, 2015). Indeed, communication between these disparate structures is essential for the proper encoding and retrieval of episodic memories (Simons and Spiers, 2003; Preston and Eichenbaum, 2013). Conceptually, the mPFC may be directing the retrieval of episodic memories from the HPC, based on the current context (Navawongse and Eichenbaum, 2013). In other words, information relayed from the mPFC to the HPC aids in guiding both memory acquisition and retrieval, while in turn, the HPC sends signals to the mPFC to provide remembered episodes with goals, rules, and procedural representations (Morris, 2001; Dolleman-van der Weel et al., 2019).

The HPC robustly and directly projects to the mPFC, strongly targeting infralimbic (IL) and prelimbic (PL) cortices (Hoover and Vertes, 2007), yet no direct projection from the mPFC back to the HPC exists (Sesack et al., 1989; Laroche et al., 2000); although cf. Rajasethupathy et al. (2015). Instead, a small midline thalamic body, the nucleus reuniens (RE), lies anatomically interposed between the mPFC and HPC (Vertes et al., 2006, 2007). Within the RE is a population of neurons that project via axon collaterals to both the mPFC and HPC, providing a direct disynaptic link between these two important structures (Hoover and Vertes, 2012; Varela et al., 2014). The RE as such is a key node for return projections from mPFC to HPC, completing a loop circuit capable of bridging executive functioning and memory: HPC → mPFC → RE → HPC (Dolleman-van der Weel et al., 2019).

Broadly speaking, the most predominant (and widely-studied) activity-dependent oscillatory patterns in these two sites are slow oscillations (SO, ~1 Hz) and the theta rhythm (~3–12 Hz). Slow oscillations are known to nest other mnemonically-relevant activity patterns, including spindles (~12–16 Hz), beta/gamma (25–100 Hz), and sharp wave/ ripple complexes (transient, irregularly-occurring high frequency: 150–250 Hz oscillations) (Staresina et al., 2015; Oyanedel et al., 2020). The importance of these rhythms for memory processes has been demonstrated during waking (Sauseng et al., 2009; Buzsaki and Watson, 2012), as well as during offline states like sleep (Puentes-Mestril et al., 2019; Marshall et al., 2020).

Recently, we have demonstrated that the RE has an essential role in coordinating SO activity between the mPFC and HPC (Hauer et al., 2019). However, less clear is how the functionality of the mPFC inputs to the HPC *via* the RE may change as a result of ongoing shifts in brain state. Moreover, it is unclear how the role of the RE may change during activated (theta) states, and how its activity (or inactivity) may modulate cortico-hippocampal information exchange. To this end, we performed a sequence of multisite recordings, along with opto- and chemogenetic circuit perturbations in an *in vivo*, urethane-anesthetized rat preparation, while stimulating both RE and IL across SO and theta states. Urethane was chosen given how closely its mimics natural sleep, including spontaneous fluctuations between activated and deactivated electrophysiological states concomitant with physiological changes, and its stability over long-duration recordings (Clement et al., 2008; Pagliardini et al., 2013a; Ward-Flanagan and Dickson, 2019; Silver et al., 2021). Our results demonstrate that PFC-HPC communication is fundamentally different between states and that the RE has a critical role in mediating this disparity. Different circuitry is recruited as a function of the ongoing forebrain state, but both states show a rhythmical modulation of excitability dependent on the oscillatory phase of the ongoing rhythm.

## Materials and Methods

### Animals

Experiments were conducted on 22 male Sprague Dawley (SD) rats obtained from the Sciences Animal Support Services and/or Health Sciences Laboratory Animal Services of the University of Alberta with a mean (±SEM) final weight of 453.09 ± 9.40 g. Of these, 14 were used for the electrical or optical stimulation of the RE, cingulum bundle (CB), and PFC; and 8 were used for the chemogenetic inhibition of the RE. Some of these data (*n* = 8 rats for DREADDs experiments; *n* = 3 for RE and CB optogenetic stimulation) were used in a prior study from our laboratory (Hauer et al., 2019), while the rest (*n* = 11) constitute entirely original experiments. The only reasons for animals to be excluded from analyses were (1) lack of appropriate anatomical expression of the virus (also verified by lack of functional manipulations of the RE and thus lack of influence in HPC recordings); (2) missed placements (of either stimulation and/or recording electrodes and/or optic fiber); (3) lack of sufficient data across both theta and SO states within a given experiment. All animals were provided with food and water *ad libitum* and were maintained on a 12 h light/dark cycle, with lights on at 7:00 a.m. All procedures conformed to the guidelines of the Canadian Council on Animal Care (CCAC) and were approved by the Biological Sciences and/or Health Sciences Animal Policy and Welfare Committees (AUP 092 and AUP 461) of the University of Alberta.

### Materials

Bipolar recording electrodes with tip length separation between 0.4 and 0.9 mm were constructed using a Teflon-coated stainless steel wire (bare diameter 125 μm; A-M A-M Systems, Sequim, WA). We also used a linear 16-contact (100 μm separation) multiprobe arranged in a vertical linear array (U-probe, Plexon Inc., Dallas, TX, USA) to assess the spatial profile field potential recordings in the HPC.

One primary viral vector was used for optogenetic experiments: an adeno-associated virus (AAV, serotype 2/2), expressing a channelrhodopsin-2 variant (ChR2/H134R). The vector was conjugated with an enhanced yellow fluorescent protein (EYFP), and driven by the synapsin promoter (hSyn-ChR2-EYFP). They were produced, characterized, and titrated at the University of North Carolina Virus Vector Core Facility, Chapel Hill, NC, USA (ChR2: 3.9 × 10^12^ molecules ml^−1^).

Chemogenetic experiments also used an AAV vector (serotype 2/5) that was also driven by the same synapsin promoter. However, the vector expressed a Gi-coupled designer receptor exclusively activated by its designer drug (DREADD; hM4Di) and was conjugated with both the mCitrine fluorescent protein and a human influenza hemagglutinin (HA) tag (hSyn-hM4Di-HA-mCitrine; 3.5 × 10^12^ molecules ml^−1^; UNC Virus Vector Core Facility).

Additionally, chemogenetic control experiments were conducted by using a virus with the same promoter (hSyn) and AAV serotype (5) that was coupled only to a fluorescent vector, without any opsin or DREADD (hSyn-mCherry; UNC Virus Vector Core Facility).

### Procedures

#### Viral Injections

The rats were initially anesthetized in a sealed chamber with gaseous isoflurane (4% induction, 1.5% maintenance, in 100% O_2_). After the loss of righting reflexes, the rats were given an intraperitoneal injection of a ketamine/xylazine cocktail (90 and 10 mg/kg, respectively; Bimeda-MTC; Animal Health Inc., Cambridge, ON, Canada; and Rompun; Bayer Inc., Mississauga, ON, Canada). Supplemental doses (10% of original dose) of the ketamine/xylazine cocktail were administered as required to maintain a surgical anesthetic plane. The body temperature was maintained at 37°C following anesthesia using a homeothermic monitoring system (Harvard Apparatus, Holliston, MA, USA).

The rats were placed into a stereotaxic apparatus (Model 900, David Kopf Instruments, Tujunga, CA, USA) and using aseptic techniques, were prepared for intracranial injections. A single incision was made along the midline of the scalp, and the skin flaps were pinned back. The skull was leveled by adjusting lambda and bregma to be in the same horizontal plane. Holes were drilled in the skull at pre-determined coordinates from stereotaxic rat atlas (Paxinos and Watson, 1998).

Micropipettes (tip diameter, 30 μm) loaded with either hSyn-ChR2-EYFP (optogenetic experiments), hSyn-hM4Di-HA-mCitrine (chemogenetic experiments), or hSyn-mCherry (control experiments) were attached to a holder (EHW-2MS; A-M Systems Inc., Carlsborg, WA, USA) and lowered using a micro-positioner into the brain. The injections targeted the midline of the nucleus RE thalami (AP −2.0; ML +1.9 mm) at an angle 16° oblique to the vertical line to avoid the midline sinus and advanced 6.8 mm from the brain surface (infusion volume 400 nl). The injections were made using a micro-injector (PMI-100; Dagan, Minneapolis, MN, USA) connected *via* tubing (PVC, 2.79 × 4.5 mm; Gilson Inc., Middleton, WI, USA) to the holder, using a pressure of 40 psi and 15 ms pulse length, at a rate of approximately 100 nl/min. Micropipettes were left in place for 7–10 min following the injection to allow for the adequate diffusion of the virus, and to prevent the unintended back travel of the injected solution up the pipette track.

Following the injection procedures, the scalp was then sutured, and the rats were given 0.5 ml of the local anesthetic bupivacaine (5 mg/ml s.c.) around the incision site. The animals were provided with pain medication (meloxicam, 1–2 mg/kg in oral suspension; Boehringer Ingelheim Vetmedica, Ingelheim, Germany) over a 24 h period post-surgery. Food and water were provided *ad libitum* and animals were allowed to recover for 3 weeks before acute experimentation (see below). Neither the viral injection nor the surgical procedures produced any observable long-term issues.

#### Acute Urethane Anesthesia and General Experimental Procedures

For acute anesthetized recordings, the rats were initially anesthetized in a gas chamber with isoflurane in medical oxygen (4% induction, 1.5% maintenance). A catheter was inserted into the femoral vein, and isoflurane was discontinued. General anesthesia was obtained by slow (~0.03–0.08 ml/min) incremental administrations of urethane (0.4 g/ml) *via* the catheter (final dose across all rats: 1.33 ± 0.05 g/kg). Urethane was chosen because it promotes an unconscious state that closely mimics the typical dynamics present during natural sleep, both in terms of brain state alternations as well as in terms of their typical physiological correlates (Clement et al., 2008; Pagliardini et al., 2013a).

The rats were placed back into the stereotaxic apparatus and once again the cranium was exposed by making a single long incision along the scalp and pinning back the skin flaps. As before, the skull was leveled by adjusting lambda and bregma to be in the same horizontal plane. The body temperature was maintained at 37°C using the same homeothermic monitoring system. Intracranial implantations were made using pre-determined coordinates from stereotaxic atlas, using bregma as a landmark (Paxinos and Watson, 1998).

In all experiments, bipolar electrodes for recording local field potentials were positioned in the PFC [AP + 3.2; ML 0.7; DV (tip of long electrode) −1.1 to −1.8 mm], and were also placed in the HPC, straddling the pyramidal layer of CA1 to maximize the amplitude of the theta recording (AP −5.5; ML −4.5 mm; DV −2.2 to −3.2 mm). These electrodes were cemented in place using dental acrylic and jeweler's screws fastened into the skull. Local field potentials from bipolar wire electrodes were amplified in differential mode at a gain of 1,000 and filtered between 0.1 and 500 Hz using a differential AC amplifier (Model 1700, A-M Systems Inc.).

#### Photostimulation

An optic fiber (tip diameter 200 μm) connected to a 473 nm laser (Laserglow Technologies, Toronto, ON, Canada), calibrated to deliver light at 10–12 mW, was positioned in order to deliver light at intracranial locations. Photostimulation events were driven by a pulse stimulator (Model 2100; A-M Systems Inc.) connected to the laser power supply as well as to the analog-to-digital board and PC acquiring data to mark each event (see below).

##### Nucleus Reuniens Stimulation Procedures

In addition to bipolar local field potential recordings of the PFC and HPC, we also used the linear multiprobe in the contralateral HPC (AP −5.5; ML +4.5; DV −3.3 to 4.5 mm), which was positioned in order sample activity throughout the vertical extent of CA1. Importantly, for monitoring the effects of RE and mPFC stimulation and/or inhibition, the intermediate HPC was consistently targeted using the linear probe given the prominent projection patterns from RE to this septo-temporal region of the HPC (Hoover and Vertes, 2012). Local field potentials from the multiprobe were referenced to stereotaxic ground, passed through a unity gain headstage, and then amplified at a gain of 1,000 and filtered between 0.1 and 500 Hz (X1000: Plexon, Dallas, TX, USA). Signals were digitized at a sampling frequency of 1,000 Hz using a Digidata 1440A analog to digital board (Molecular Devices; San Jose, CA) connected to a PC running Axoscope (Molecular Devices). The final depth of the probe was determined using the well-established electrophysiological profile of theta field activity (Bland and Bland, 1986; Buzsaki, 2002). The position of the multiprobe was histologically confirmed in every experiment by analyzing its track in relation to recorded field activity.

The optic fiber was first positioned above the RE (AP −2.0; ML +1.9; DV −6.4 mm) at an angle 16° oblique to the vertical line. Following sufficient optic stimulation (described below), the optic fiber was removed and re-positioned to target the CB (AP −2.5; ML +2.7 mm), angled at 40° oblique to the vertical line and advanced 2.6–3.4 mm from the brain surface. Evoked potentials were produced using 10 ms laser pulses delivered every 5 s to the RE and then subsequently, the CB, and were averaged over 32–64 trials. Stimulation trains were delivered during equivalent brain states, specifically during either clear deactivated periods characterized by ongoing high power in the 1 Hz signal, or while prominent theta (~4 Hz) activity could be observed in the HPC with concomitant low voltage, higher frequency activity in the PFC. Stimulation during particular states was confirmed by monitoring the ongoing brain state when delivering stimulation trains; subsequently by both visual and spectral examination of individually recorded sweeps. For the purposes of assessing phase preference, optical stimuli were also delivered every 5 s during continuous field recordings, with the goal of delivering stimuli at random phases of the oscillatory cycle.

##### Medial Prefrontal Cortex Stimulation

In a subset of animals (*n* = 18), we employed either a single or paired pulse stimulation paradigm in the mPFC sites, with the goal of evoking HPC potentials. The surgical preparation was identical to that employed for RE stimulation (see above), except that instead of targeting an optic fiber over RE or CB, a bipolar stainless steel (0.0″ bare, 0.11″ Teflon coated) stimulating electrode was lowered into the infralimbic (IL) zone (AP +2.8 to +3.2; ML +0.7 to +1.1; DV −4.0 to −4.5 mm). Following the mPFC paired pulse stimulation paradigm used by Gemmell and O'Mara (2000), 100–500 μA biphasic current pulses 0.5 ms in duration were delivered with a 30 ms inter-stimulus interval, every 8 s using a constant current stimulator (Model 2100; A-M Systems Inc.). In some instances, current pulses were also delivered with a 50 ms inter-stimulus interval, or as single, stand-alone pulses. Stimulation epochs were averaged over 32 trials and were always delivered during a consistent brain state, either theta or SO. Epochs containing trials with electrophysiological artifacts or with sudden, brief state transitions were removed from the analyses on an individual, trial-by-trial basis. Similar to the optical stimuli delivered to RE and CB during continuous recordings, electrical pulses were delivered to the IL for the purposes of assessing phase preference. Single pulse stimuli were delivered every 8 s during continuous field recordings, with the goal of delivering stimuli at completely random phases of the oscillatory cycle.

##### Nucleus Reuniens Chemogenetic Inactivation

In 8 experiments with IL stimulation, rats had been pre-treated to express either hSyn-hM4Di-HA-mCitrine or hSyn-mCherry in the RE via our viral injection procedures as described above. Subsequent to baseline evoked potential analysis, and after a suitable period of spontaneous recordings, the DREADD agonist Clozapine N-oxide (CNO; Cayman Chemical, Ann Arbor, MI, USA) was administered *i.p*. at a dose of 3 mg/kg (MacLaren et al., 2016). The activity was then recorded for ~2 more hours, followed by the same IL stimulation paradigm described above. Evoked potentials were performed at least 30 min post *i.p*. CNO injection to ensure adequate time for the ligand or its metabolites to enter the brain (Whissell et al., 2016). This allowed for characterization of the HPC response evoked by IL stimulation before and after RE inactivation. We could also then compare the evoked response profile in the HPC with an intact vs. inactive RE within the same animals and recording period.

### Perfusion and Histology

Following experimental recordings, 5 s direct current pulses of 1 mA using an isolated current pulse generator (Model 2100; A-M Systems Inc.) were passed through bipolar recording and stimulating electrodes to generate small electrolytic lesions at their tips. These lesions allowed for subsequent verification of recording and stimulation sites. Rats were then transcardially perfused with 0.9% saline and 4% paraformaldehyde in saline (Fisher Scientific, Toronto, ON, Canada). The brain was then removed and placed into a 4% formalin and 20% sucrose solution for at least 48 h. The brains were flash-frozen using compressed carbon dioxide (CO_2)_ and sectioned with a rotary microtome (1320 Microtome; Leica, Vienna, Austria) at a width of 60 μm. The tissue was counter-sectioned, with one-third of the sections being mounted on gelatin-coated microscope slides for subsequent thionin staining; another third being mounted on slides and immediately covered using a fluorescence preserving reagent and mounting medium (FluorSave; EMD Millipore, Darnstadt, Germany); and a third of the tissue saved for immunohistochemistry for detection of specific neuronal markers.

Immunohistochemistry was performed according to the following protocol. Free-floating sections were rinsed three times using phosphate-buffered saline (PBS) and incubated with 10% normal donkey serum (NDS) and 0.3% Triton X-100 for 60 min to reduce non-specific staining and increase antibody penetration. Sections were left to incubate overnight with primary antibodies diluted in PBS containing 1% NDS and 0.3% Triton X-100 at room temperature. Primary antibodies used detected: green fluorescent protein (GFP; dilution 1:1000; raised in chicken; Aves Labs, Tigard, OR, USA); red fluorescent protein (mCherry; dilution 1:800; raised in rabbit; Millipore); human influenza hemagglutinin (HA; 1:800; raised in rabbit; Cell Signaling Technology, Danvers, MA, USA); and neuronal nuclear marker (NeuN; 1:800; raised in mouse; Millipore). The following day, the tissue was again washed three times with PBS, incubated with secondary antibodies conjugated to the specific fluorescent proteins in each viral construct (Cy2-conjugated donkey anti-chicken; Cy3-conjugated anti-rabbit; Cy5-conjugated anti-mouse; 1:200; Jackson ImmunoResearch, West Grove, PA, USA) diluted in PBS and 1% NDS for 2 h. The sections were again washed three times with PBS, mounted, and coverslipped with Fluorsave (EMD Millipore). Microscopic inspection of tissue was used to verify electrode recording loci, optic fiber tracks, and expression of viral constructs using a Leica DM5500B fluorescent microscope, Leica Microsystems Inc., Concord, Ontario, Canada.

### Data Processing and Analysis

All signals were acquired with Axoscope 10.6 (Molecular Devices) and were first examined visually to choose data segments for further analyses. Computation and analyses were conducted using custom-written code, as well as the Circular Statistics Toolbox (Berens, 2021) in MATLAB (version R2015b, Mathworks; Natick, MA). Analyses were further processed with CorelDRAW X6 (Corel; Ottawa, Ontario, Canada). Data analyses are highly similar to those described in Hauer et al. (2019) and briefly included the following: zero phase delay digital filtering, evoked potential averaging, power and phase profile and spectral analyses, coherence, current source density, single- and dual-channel spectra, auto- and cross-correlations, and circular (Rayleigh) statistics. Two-tailed paired *t*-tests are performed throughout, given the within-subjects design of our experiments.

#### Field Recordings

Autopower, crosspower, coherence, and cross-phase spectra were computed and visualized for sets of field signals. The spectra were estimated using a series of 6-s-long, Hanning-windowed samples with 2 s overlap using Welch's periodogram method. Power spectrograms (Wolansky et al., 2006; Whitten et al., 2009; Hauer et al., 2019) were calculated with a sliding window procedure, enabling discrete spectra to be computed and visualized at specific time points across the duration of the recording. These windows were 30 s in duration, slid across the file in 10 s increments. These discrete spectra were then visualized and analyzed as described above. Spectral profiles from multiprobe recordings were created in the same manner for the activity recorded at each channel of the multiprobe, except that each channel was compared against a fixed (either PFC or HPC) bipolar reference, enabling extraction of power, phase, and coherence information at spectral peak frequencies for both SO and theta states. The spatial locations of the multiprobe channels were estimated based on the well-described theta power profile (Bland and Bland, 1986; Buzsaki, 2002; Wolansky et al., 2006), with the phase reversal point corresponding to the interface between *stratum pyramidale* and *stratum radiatum*, and the maximal theta channel located at *stratum lacunosum moleculare* (SLM).

#### Current Source Density Analysis

The CSD analysis was performed on both spontaneously recorded (gap-free) field samples, or on evoked potential sweeps and averages recorded using the multiprobe. Briefly, CSD was computed by estimating the second spatial derivative of adjacent multiprobe voltage traces, following the assumptions of Freeman (1975), Rodriguez and Haberly (1989), and Ketchum and Haberly (1993). An advantage of this analytical technique is that it eliminates potential contamination from volume conducted fields at distant sites because it estimates the volume density of the net current entering or leaving the extracellular space at a particular site (Buzsaki et al., 2012). Spectral (particularly power and coherence) estimates of the CSD were computed as described above, comparing the CSD of individual multiprobe channels against each other, or against fixed PFC or HPC bipolar electrodes. This technique enabled visualization and analysis of the magnitude of current sinks and sources as a function of space.

#### Slope and Hilbert Phase Analysis

To compare the degree of excitatory input across states, we first examined the negative slopes of the evoked field excitatory postsynaptic potentials (EPSPs) due to the stimulation of either IL or RE. Using ClampFit 10.7 (Molecular Devices), we manually defined the beginning and end points of the initial negative-going portion of the evoked potential to compute the maximum slope on an individual, sweep-by-sweep basis, as well as on average. For determining the phase value at which the evoked potential was triggered, we computed the Hilbert transform of the ongoing band-passed rhythm (0.5–1.5 Hz for SO and 3–6 Hz for theta). This analytic approach enabled the computation of instantaneous amplitude and phase for each type of signal (Le Van Quyen et al., 2001; Bruns, 2004). Phase values were standardized to their sine equivalents by adjusting resultant cosine values by 90° so that positive-going zero crossings were at phase 0°, 90° the positive peak, 180° the negative-going zero crossing, 270° the negative peak, and 360° the final positive zero crossing and thus, the complete cycle. This allowed us to define the exact phase at which stimulation occurred and then later subcategorize it as occurring during either the rising (270–90°) or falling (90–270°) phase of the ongoing oscillation.

To assess the relationship between evoked potential slope and the neocortical and hippocampal field, we subdivided evoked potentials across cycles into 20 (18°-wide) bins according to the phase of the field cycle at which they occurred. Since individual slope values could be variable, especially considering the large amplitude of ongoing potentials, we applied an adjacent-averaging (3 bin-wide) smoothing function to these values. This provided an estimate of the relative magnitude of evoked potential responding as a function of the ongoing oscillation during SO vs. during theta, for both IL and RE stimulation. Rayleigh statistics for circular data were used to statistically evaluate the significance of average phase preference on an experiment-by-experiment basis (Zar, 1999). Only experiments where stimuli were delivered with a relatively uniform distribution across all phases of the cycle (i.e., across all 20 phase bins) were included.

## Results

### Histological Findings

**Figure 1:**
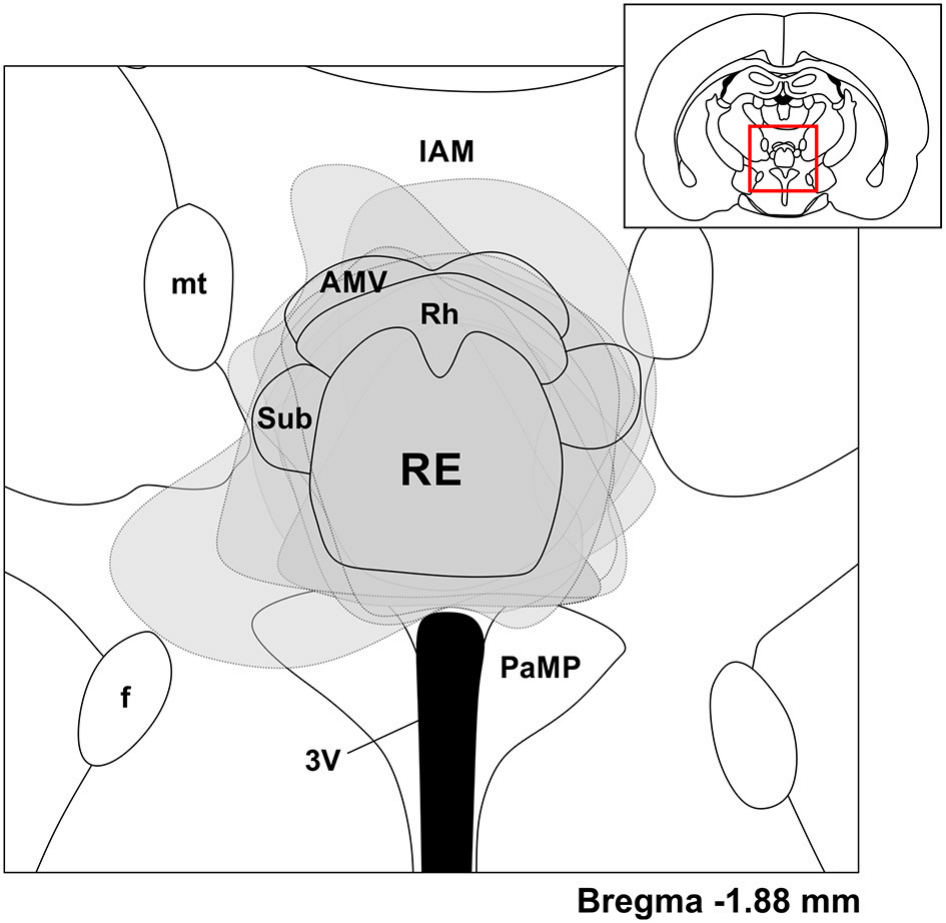
Viral expression was largely confined to the nucleus reuniens (RE). Coronal schematic depiction of the ventral midline thalamus according to a rat brain atlas (Paxinos and Watson, 1998). Gray dashed lines and areas correspond to viral expression across individual experiments, transparent and overlaid on the same schematic, based on a position of −1.88 mm from bregma. Inset, Coronal schematic of the relative location (red box) of the expansion. IAM, interanteromedial nucleus of thalamus; mt, mammillothalamic tract; AMV, anteromedial thalamic nucleus, ventral part; Rh, rhomboid nucleus of thalamus; Sub, submedius nucleus of thalamus; RE, reuniens nucleus of thalamus; f, fornix; 3V, third ventricle; PaMP, paraventricular hypothalamic nucleus, medial parvicellular part.

In every experiment, we confirmed the location of all recording and stimulation electrodes, as well as the positioning of the optic fiber. Using immunohistochemistry, we also confirmed the location of viral vector expression (Figure 1). Bipolar electrodes were successfully placed in their respective positions (mPFC or HPC) in all cases. All linear multiprobe tracks were localized to CA1, spanning from *stratum pyramidale* through SLM, and into the molecular layer of dentate gyrus (DG). Localized viral expression was largely confined to the RE, with occasional expression in the adjacent rhomboid, submedius, and ventral anteromedial nuclei (Figure 1). Any experiments in which viral expression was not localized appropriately to the RE showed a lack of electrophysiological responsiveness to optogenetic stimulation and were excluded from any further analysis. The latter finding additionally suggests that any effects observed in response to laser application were optogenetically mediated, and were not caused by heat or light-induced artifacts. Placement of the optic fiber was also verified in the immuno-labeled slices, and in all RE-targeting experiments, was positioned just dorsal to the RE, approximately on the midline. Similarly, in experiments where CB stimulation was performed, the optic fiber tip was just dorsolateral relative to the CB at an angled approach. The tip of the stimulation electrode targeting IL was found to consistently be located within IL, just ventral to the prelimbic cortex and above dorsal peduncular cortex, and always medial relative to the basal ganglia.

### Determination of Forebrain State

**Figure 2:**
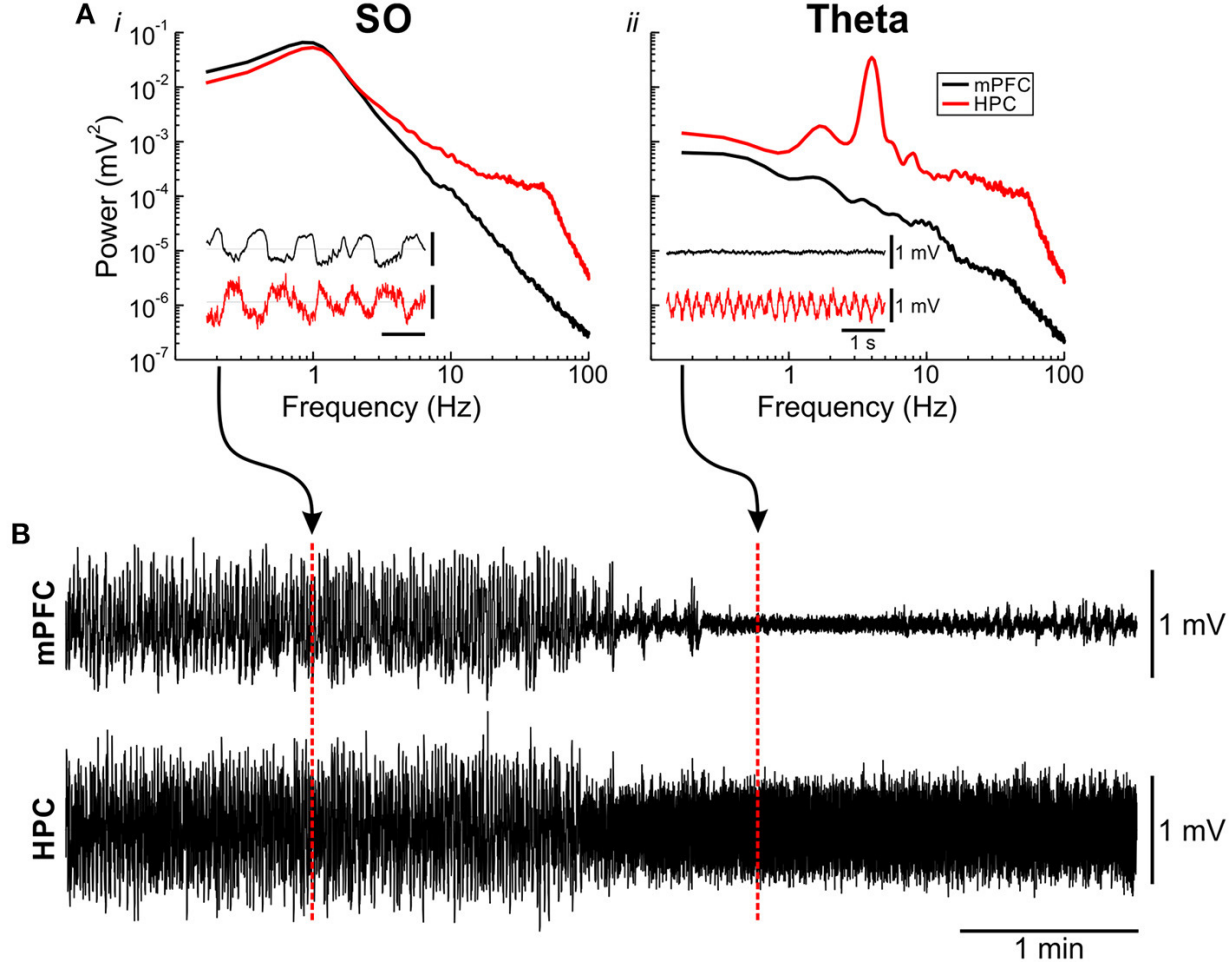
Urethane-anesthetized animals display spontaneous alternations of forebrain state. **(A)** Power spectra and raw local field traces (inset) of the slow oscillation (SO) [deactivated; **(Ai)**] and theta [activated; **(Aii)**] states observed under urethane anesthesia. During the SO state **(Ai)**, large-amplitude ~1 Hz fluctuations can be seen in the medial prefrontal cortex (mPFC) (black) and hippocampus (HPC) (red) field, with spectral power maxima at both sites at ~1 Hz. During the theta state **(Aii)**, low voltage, fast activity is apparent in mPFC (black) with a prominent ~4 Hz theta rhythm obvious in the HPC (red), which is also reflected in the power spectrum. (**B)** Continuous local field traces of a spontaneous transition from a deactivated (SO) to an activated (theta) state. Positions from where the inset exemplar traces were taken from are highlighted with dashed red lines.

Across all experiments and stimulation trials, we paid close attention to the ongoing forebrain state (Figure 2). Deactivated (SO) periods under urethane anesthesia are characterized by high power in the 1 Hz signal across both cortical and hippocampal sites (Figure 2A). Conversely, during activated (theta) states, cortical regions display lower voltage and higher frequency activity, while the HPC shows a prominent theta (~4 Hz) rhythm (Figure 2A). These transitions are spontaneous and periodic, occurring every ~10–12 min (Clement et al., 2008). Both states are obvious when examining the raw field activity (Figure 2B), as well as with spectral methods to assess power across frequencies (Figure 2A). As such, determination across individual stimulation trials was performed first on visual inspection of the raw traces, followed by any necessary spectral validation. Each trace was manually confirmed on a one-by-one basis to be within the appropriate state.

### Nucleus RE Stimulation Produces a Stronger Hippocampal Response During Deactivated States

**Figure 3:**
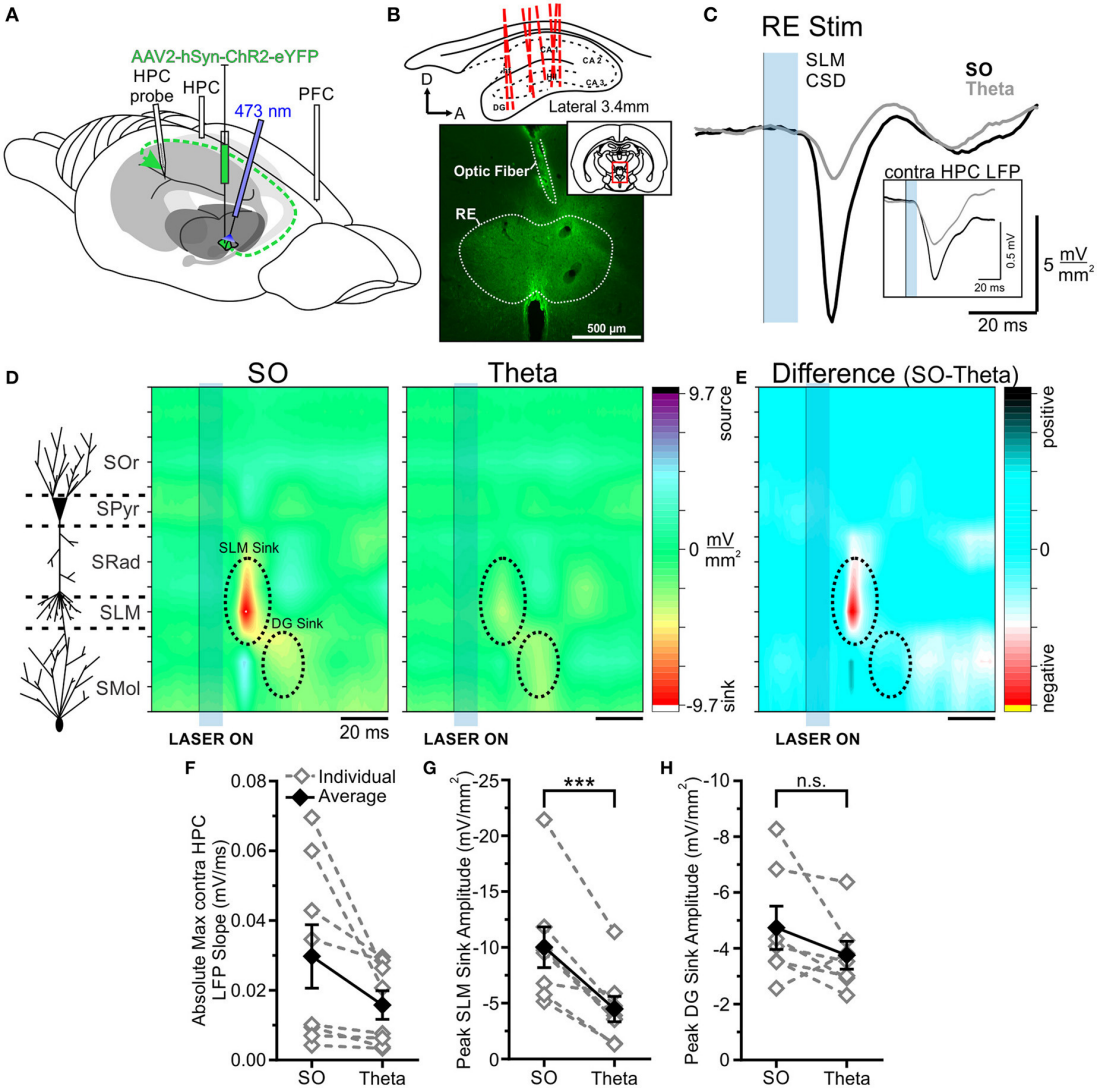
Optogenetic stimulation of RE evokes a much larger response in HPC during SO. **(A)** Schematic illustration of the recording, injection, and stimulation sites. Schema modified from Amaral and Witter (1995). **(B)** Top, Schematic sagittal illustration of all multiprobe tracks through HPC. Bottom, Representative coronal tissue section depicting the expression of hSyn-ChR2-EYFP virus localized largely to RE, with optic fiber track positioned dorsal to RE. Inset, Coronal schematic of the relative location (red box) of the image. **(C)** Current sink/source density traces evoked in stratus lacunosum moleculare (SLM) following optogenetic stimulation (10 ms pulse at 473 nm wavelength; blue rectangle) of RE during SO (black line) and theta (gray line), averaged over 64 trials. Stimulation of RE during SO produces a much larger current sink in HPC than does stimulation during theta. Inset, Local field potentials recorded from the contralateral HPC following the same optogenetic stimulation of RE during SO (black line) and theta (gray line), showing a larger response during SO. **(D)** Left, Schematic depiction of HPC cell lamina as determined by theta profile. Color contour plot of current source density (CSD) values just before and following 10 ms optogenetic stimulation of RE (black line and blue rectangle) during SO (middle) and during theta (right). RE stimulation produces a large current sink centered around SLM (with a corresponding current source in DG) which is of much greater magnitude during SO compared to during theta. A later DG sink is also evoked by RE stimulation, which does not significantly differ in amplitude between states. CSD scales for both contour plots are identical, from −9.7 to 9.7 mV/mm^2^. **(E)** Difference contour plot created by subtracting the theta CSD [**(D)**, right] from the SO CSD [**(D)**, middle] in the same animal, illustrating that the difference between the two states is largely confined to the SLM sink. The scale of difference contour plot is −6.4–6.4 mV/mm^2^. **(F)** Absolute value of the maximum local field potential slope evoked in contralateral HPC following optogenetic stimulation of RE averaged over 32–64 trials, for individual rats (gray, hollow diamonds, and dashed gray lines) and on average (black, filled diamonds, and solid black line). Stimulation during SO evokes a slope of greater magnitude than stimulation during theta in every case, although this difference is not significant overall (*p* = 0.057). **(G)** Peak SLM sink amplitude evoked by optogenetic stimulation of RE during SO and theta in the same individual animals (gray, hollow diamonds, and dashed gray lines) and on average (black, filled diamonds, and solid black line). A significantly larger sink at SLM is evoked during SO than during theta. **(H)** Peak DG sink amplitude evoked by optogenetic RE stimulation across both states, in individuals (gray, hollow diamonds, and dashed gray lines) and on average (black, filled diamonds, and solid black line), showing no significant difference across states. Error bars represent SEM. ***p < 0.001. SOr, Stratum oriens; SPyr, stratum pyramidale; SRad, stratum radiatum; SLM, stratum lacunosum-moleculare; SMol, stratum moleculare.

Following viral infection of the RE with hSyn-ChR2-EYFP in eight rats, optical pulses were delivered to the RE (Figure 3). To confirm the observed effects were not due to off-target expression in other nuclei, we additionally stimulated the isolated primary efferent from RE to CA1 in the CB (see below). Evoked potentials were averaged over between 32 and 64 individual stimulations, delivered within each state. Our primary index for brain state was determined by the neocortical electrode, but the HPC activity was recorded as an additional confirmation of state.

As we have previously shown (Hauer et al., 2019), the opto-stimulation of the RE evoked a prominent negative-going potential peaking at a latency of 24.75 ± 0.73 ms from laser onset in both bipolar and multiprobe recordings from the HPC. In multiprobe recordings, the maximum negativity was observed at the approximate level of the SLM. Current source density profiles confirmed that the shortest latency sink (peaking at a similar latency of 22.94 ± 0.60 ms) was indeed centered at the SLM.

When comparing responses across states we observed a consistently larger evoked response (in all 8 of 8 experiments) when light pulses were delivered during ongoing slow oscillation (deactivated) states as compared with theta (activated) states. Stimulation during SO states produced a significantly larger current sink (−10.03 ± 1.82 mV/mm^2^) that was maximal at SLM than did stimulation during theta states (−4.48 ± 1.13 mV/mm^2^; *p* = 0.00060, two-tailed paired *t*-test; Figures 3D,G). Similarly, the optogenetic activation of RE during SO evoked a larger local field potential (averaged over 32–64 stimulation trials per animal) in the contralateral HPC bipolar recording than did excitation during theta. This was a consistent effect in every animal, however, the magnitude was not statistically significant overall (SO: 0.030 ± 0.0091 mV/ms; theta:0.016 ± 0.0041 mV/ms; *p* = 0.057, two-tailed paired *t*-test; Figures 3C,F).

No significant difference in latency to the maximal SLM sink was observed across states (SO: 23.63 ± 0.96 ms; theta: 22.25 ± 0.70 ms; *p* = 0.29, two-tailed paired *t*-test). As such, the overall pattern of current sinks and sources was otherwise consistent across both states (Figure 3E), and with previous literature (Dolleman-Van der Weel et al., 1997; Dolleman-van der Weel et al., 2017; Hauer et al., 2019; Vu et al., 2020). In 7 rats, we compared the somewhat later evoked sink in DG across states, and all but one showed a greater sink magnitude during SO states (−4.74 ± 0.77 mV/mm^2^) compared with during theta (−3.76 ± 0.50 mV/mm^2^; Figure 3H). However, this difference was not significant overall (*p* = 0.15, two-tailed paired *t*-test). Unlike the SLM sink, however, the peak of the DG sink occurred slightly later during SO (39.29 ± 1.66 ms) than during theta (35.57 ± 0.81 ms; *p* = 0.031, two-tailed paired *t*-test). As such, the primary difference between the states was the amplitude of the current sink at SLM, where RE projections synapse on distal apical dendrites of CA1 pyramidal cells (Herkenham, 1978; Figure 3E).

**Figure 4:**
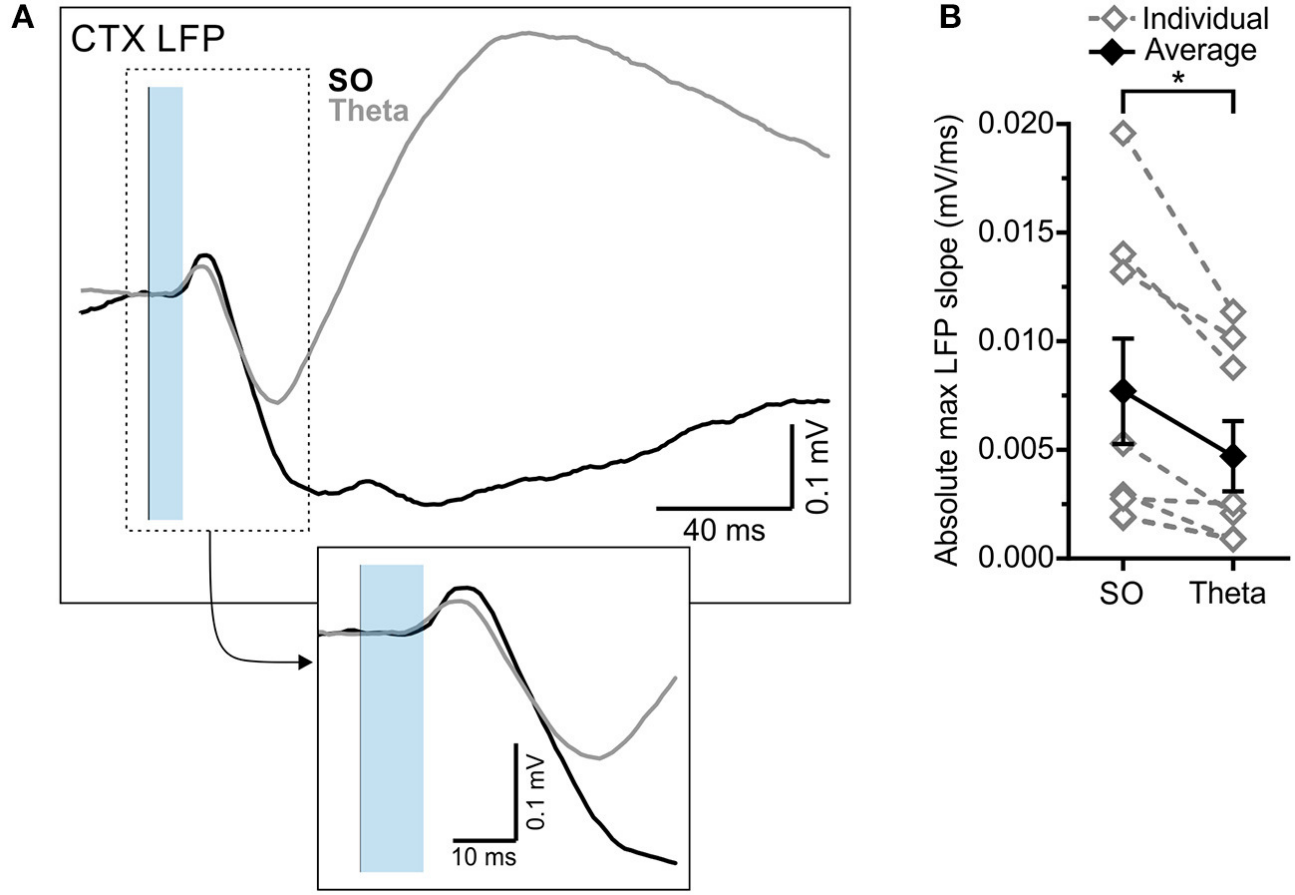
RE-evoked cortical field potentials are modulated by state. **(A)** Cortical local field potential evoked by 10 ms optogenetic stimulation of RE (blue transparent rectangle), averaged over 64 trials in both SO (black) and theta (gray) states. Inset: Magnified representation of the box in the main panel, highlighting the initial slope of the evoked response. **(B)** Absolute maximum slope of the initial cortical potential in **(A)** across both states, in individuals (gray, hollow diamonds, and dashed gray lines) and on average (black, filled diamonds, and solid black line). *p < 0.05.

The optogenetic stimulation of the RE also produced an evoked potential in the frontal cortex, which was similarly modulated by state (Figure 4). Stimulation during SO states evoked a significantly larger absolute maximum evoked potential slope than did stimulation during theta (SO: 0.0077 ± 0.0024 mV/ms; theta:0.0047 ± 0.0016 mV/ms; *p* = 0.015, two-tailed paired *t*-test; Figures 4A,B). The latency to the peak negativity was not significantly different between states (SO: 43.00 ± 7.54 ms; theta: 37.00 ± 3.79 ms; *p* = 0.37, two-tailed paired *t*-test; Figure 4A).

**Figure 5:**
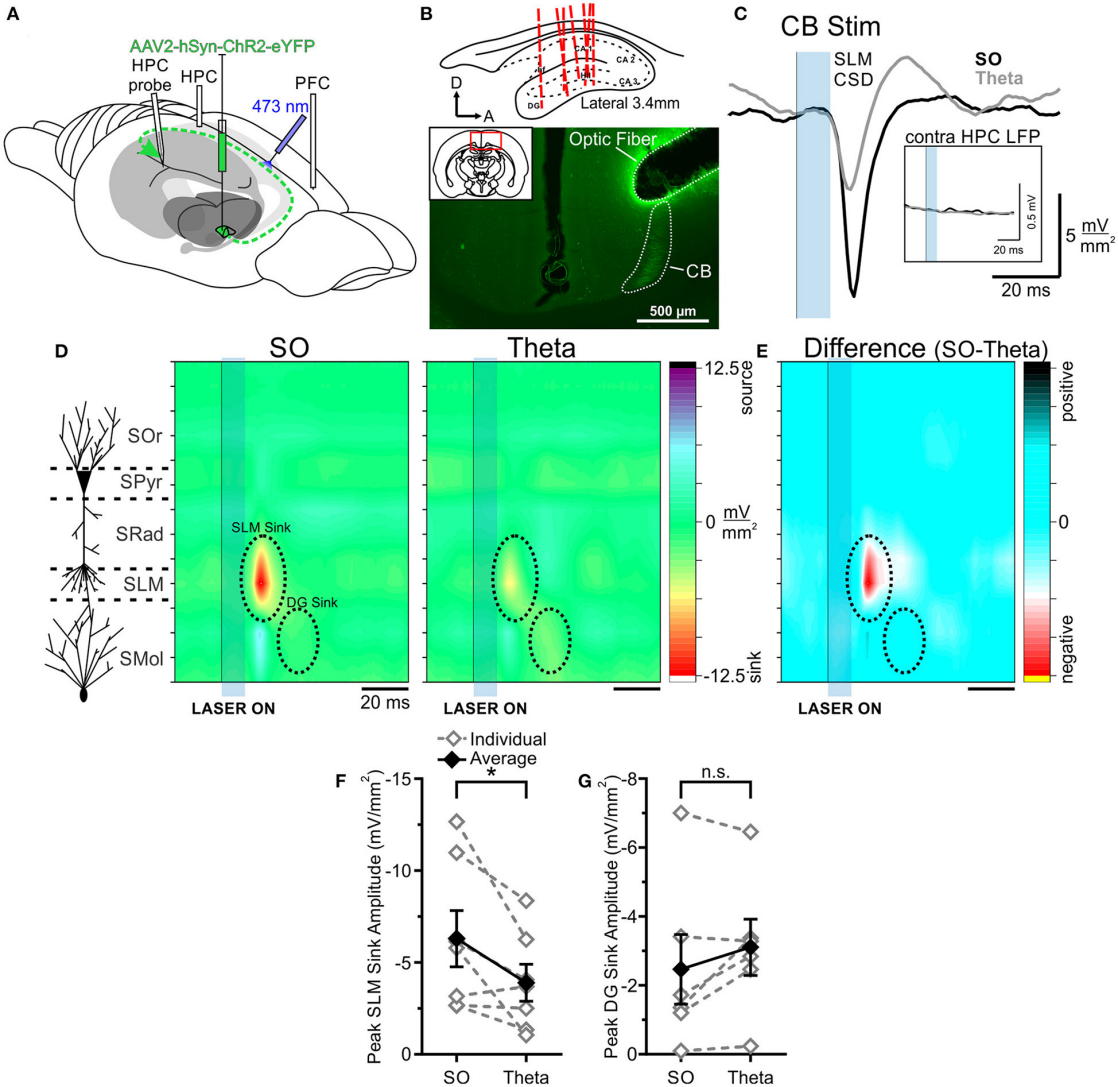
Optogenetic stimulation of the cingulum bundle (CB) evokes a larger response in HPC during SO. **(A)** Schematic illustration of the recording, injection, and stimulation sites. Schema modified from Amaral and Witter (1995). **(B)** Top, Schematic sagittal illustration of all multiprobe tracks through HPC. Bottom, Representative coronal tissue section depicting hSyn-ChR2-EYFP viral expression in CB following a single injection in RE, with optic fiber track positioned dorsolaterally to CB. Inset, Coronal schematic of the relative location (red box) of the image. Histology corresponds to the same animal whose data are depicted throughout this figure. **(C)** Current sink/source density traces evoked in SLM following optogenetic stimulation (10 ms pulse at 473 nm wavelength; blue rectangle) of CB during SO (black line) and theta (gray line), averaged over 64 trials. Stimulation of CB during SO produces a much larger current sink in HPC than did stimulation during theta. Inset, Local field potentials recorded from the contralateral HPC following the same optogenetic stimulation of CB during SO (black line) and theta (gray line). There is a total lack of response in the contralateral HPC regardless of state given the ipsilateral projection pathway of RE-to-HPC via the CB. **(D)** Left, Schematic depiction of HPC cell lamina as determined by theta profile. Color contour plot of CSD values just before and following 10 ms optogenetic stimulation of CB (black line and blue rectangle) during SO (middle) and during theta (right). CB stimulation produces a large current sink centered around SLM (with a corresponding current source in DG) which is of greater magnitude during SO compared to during theta. A later DG sink is also evoked by CB stimulation that does not significantly differ in amplitude between states. The overall pattern of responding is effectively identical to that evoked by RE stimulation. CSD scales for both contour plots are identical, from −12.5 to 12.5 mV/mm^2^. **(E)** Difference contour plot created by subtracting the theta CSD [**(D)**, right] from the SO CSD [**(D)**, middle] in the same animal, illustrating that the difference between the two states is largely confined to the SLM sink. The scale of difference contour is −6.8 to 6.8 mV/mm^2^. **(F)** Peak SLM sink amplitude evoked by optogenetic stimulation of CB during SO and theta in the same individual animals (gray, hollow diamonds, and dashed gray lines) and on average (black, filled diamonds, and solid black line). A significantly larger sink at SLM is evoked during SO than during theta. **(G)** Peak DG sink amplitude evoked by optogenetic CB stimulation across both states, in individuals (gray, hollow diamonds, and dashed gray lines) and on average (black, filled diamonds, and solid black line), showing no significant difference across states. Error bars represent SEM. *p < 0.05. SOr, Stratum oriens; SPyr, stratum pyramidale; SRad, stratum radiatum; SLM, stratum lacunosum-moleculare; SMol, stratum moleculare.

In 7 of the above 8 rats, we also successfully delivered optical stimulation to the cingulum bundle to activate RE efferents to the HPC. In this way, we were able to isolate and verify the RE-mediated component of this pathway, if any other surrounding thalamic nuclei were being excited by the optogenetic stimulation (Figure 5). As we have previously shown (Hauer et al., 2019), the pattern of evoked field and sink/source activity in HPC evoked by this stimulation was virtually indistinguishable from that evoked by direct RE activation (compare responses across Figures 3, 5 conducted in the same animal). Light delivery to the CB evoked a prominent negative-going potential peaking at a latency of 18.43 ± 0.83 ms in multiprobe recordings from the HPC. As with direct RE stimulation, the maximum negativity in multiprobe recordings was observed at the level of the SLM, and again, current source density profiles confirmed that the shortest latency sink (peaking at a similar latency of 18.00 ± 0.64 ms) was indeed centered at the SLM.

When comparing responses across states, we again observed a robust difference in the magnitude of responses of CB stimulation. As with RE, CB excitation yielded a much larger current sink during SO (−6.30 ± 1.53 mV/mm^2^) compared with during theta (−3.90 ± 1.00 mV/mm^2^; *p* = 0.042, two-tailed paired *t*-test; Figures 5D,F). As with direct RE stimulation, no significant difference in latency to the peak SLM sink was observed between states (SO: 18.29 ± 0.92 ms; theta: 17.71 ± 0.97 ms; *p* = 0.44, two-tailed paired *t*-test). As previously shown (Hauer et al., 2019), no response was observed in the contralateral HPC, consistent with the unilateral projection pattern *via* the CB (Figure 5C).

As with RE stimulation, no amplitude difference was observed in terms of the later DG sink evoked by optic CB stimulation (SO: −2.47 ± 1.01 mV/mm^2^; theta: −3.11 ± 0.82 mV/mm^2^; *p* = 0.17, two-tailed paired *t*-test; Figure 5G). Although the latency to the peak DG sink was similarly longer during SO as opposed to theta states with CB stimulation, this difference was not significant (SO: 35.50 ± 2.64 ms; theta: 31.33 ± 1.41 ms; *p* = 0.19, two-tailed paired *t*-test).

### Forebrain State Biases the Pattern of Hippocampal Responding to Medial Prefrontal Cortex Stimulation

**Figure 6:**
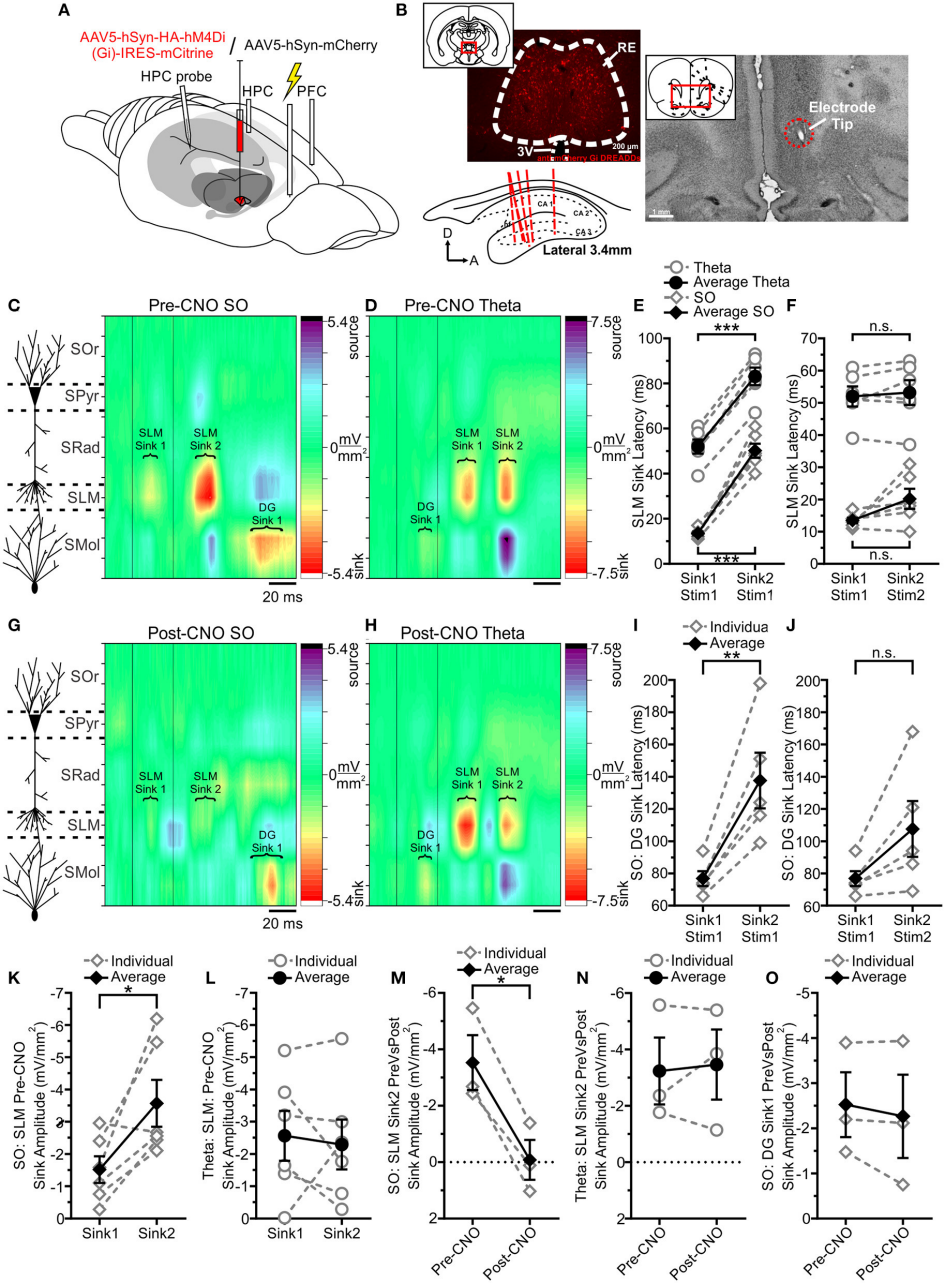
Infralimbic stimulation evokes a different pattern of responding in HPC as a function of state, which RE mediates. **(A)** Schematic illustration of the recording, injection, stimulation, and inhibition sites. Schema modified from Amaral and Witter (1995). **(B)** Originally published in Hauer et al. (2019), with modifications. Representative coronal tissue section showing expression of hSyn-hM4Di-HA-mCitrine virus localized to RE. Inset, Schematic of the relative location of the image. Top right, Representative coronal Nissl-stained tissue section showing the tip of a stimulating electrode in IL. Inset, Schematic of the relative location of the image. Bottom left, Schematic sagittal illustration of all (except one) multiprobe tracks through HPC. **(C)** Left, Schematic depiction of HPC cell lamina as determined by theta profile. Right, Color contour plot of CSD values just before and following 30 ms inter-stimulus interval paired pulse stimulation of IL. During SO, each pulse evokes a prominent current sink that is maximal around SLM, with a corresponding DG source. The second SLM sink is noticeably facilitated. A late DG sink can be observed well after the SLM sinks that correspond to each pulse. **(D)** During theta, each pulse evokes a current sink that is maximal at SLM, but with a considerably longer latency than during SO. A small initial DG sink can be observed following the first pulse, which is obscured following the second pulse by a larger current source. **(E)** Latency to peak SLM sinks relative to the first pulse across both states. The second SLM sink is maximal after a consistent latency difference (~30 ms) in both states. Latency to both maximal SLM sinks is much longer when stimulation is delivered during theta, compared with SO. **(F)** Latency to peak SLM sinks, relative to the pulse each putatively corresponds to (i.e., subtracting 30 ms from the latency to the second SLM sink). Accounting for the inter-stimulus interval demonstrates that each sink corresponds to each pulse. **(G)** Left, Schematic depiction of HPC cell lamina as determined by theta profile. Right, Chemogenetically inhibiting RE and then delivering the same paired pulse stimulation to IL produces an obviously different pattern of responding in HPC. The SLM sinks corresponding to each pulse are severely diminished, and no facilitation can be seen in the second sink as such. The late DG sink remains unaffected. **(H)** Paired pulse stimulation of IL during theta with RE chemogenetically inhibited does not change the pattern of responding in HPC. Both SLM sinks remain intact, and so too does the initial DG sink. **(I)** Latency to the peak DG sink evoked during SO when IL is stimulated, with RE intact, showing a more variable latency to the second sink. **(J)** Latency to peak DG sinks during SO relative to the pulse each putatively corresponds to (i.e., subtracting 30 ms from the latency to the second SLM sink) shows there is no significant difference in latency, indicating that each DG sink corresponds to each IL pulse. **(K)** Peak SLM sink amplitudes during SO with RE intact, showing a larger response in the second sink, indicating paired-pulse facilitation is occurring. **(L)** Peak SLM sink amplitudes during theta with RE intact, showing no difference in sink magnitude, indicating paired-pulse facilitation is not occurring. **(M)** Amplitude of the second peak SLM sink SO comparing stimulation with RE intact (pre-CNO) against stimulation while RE is chemogenetically inactivated (post-CNO). Following RE inhibition, the second SLM sink during SO is almost entirely abolished. **(N)** Amplitude of the second peak SLM sink during theta, comparing stimulation with RE intact (pre-CNO) against stimulation while RE is chemogenetically inactivated (post-CNO). There is no difference in second SLM peak sink amplitude during theta when RE is inhibited. **(O)** Amplitude of the first peak DG current sink is unchanged during SO regardless of whether RE is inactivated or not. In all panels, diamonds represent stimulation during SO; circles represent stimulation during theta; gray, hollow symbols, and dashed gray lines represent individual animals; black, filled symbols and solid black lines represent the average. *p < 0.05; **p < 0.01; ***p < 0.001.

In 6 rats, we electrically stimulated the IL zone of the mPFC, and monitored the evoked HPC potentials across deactivated and activated brain states (Figure 6). We were especially interested in the contributions of the interposed RE in this communication (Vertes, 2002; Mathiasen et al., 2019). We first verified that IL stimulation could evoke a potential in the hippocampus by monitoring field responses at both the bipolar and multiprobe sites. A short latency (20.50 ± 0.67 ms at peak) and prominent negative-going potential were observed in both hemispheres that appeared to be maximal at the level of the SLM in the linear multiprobe recordings. In order to maximize this response, we used a paired-pulse paradigm that was designed to produce strong paired-pulse facilitation at the level of the HPC. In addition, we computed the CSDs of the evoked potential responses when elicited during either SO or theta. We then assessed the pattern of IL-evoked HPC responses across deactivated and activated states, both pre- and post-CNO.

Stimulating IL yielded a remarkably distinct pattern of HPC excitation as a function of brain state. Across both states, prominent current sinks were observed at the level of SLM. However, the timing of these sinks was markedly different between SO and theta states (Figures 6C,D). During SO, the first stimulus yielded a maximal sink at SLM with a latency of 13.50 ±0.85 ms, while the second stimulus prompted another SLM sink at a latency of 20.17 ± 3.11 ms. This contrasted sharply with stimulation delivered during theta, which produced sinks at latencies of 52.00 ± 3.12 and 53.17 ± 3.87 ms following the first and second stimuli, respectively. The latency values of these two sinks from their respective stimuli were not significantly different within states (SO: *p* = 0.095; theta: *p* = 0.46, two-tailed paired *t*-tests; Figure 6F). Indeed, the differences observed in absolute latency (relative to the timing of the first stimulus in the pair, Figure 6E) are entirely attributable to the 30 ms inter-stimulus interval, with the second sink following the first by ~30 ms in both states.

A sink in the molecular layer of DG was evoked following stimulation across states as well (Figures 6C,D). During SO, a maximal sink could be observed 76.80 ± 4.66 ms following the first stimulus, with a second DG sink occurring at a more variable latency, 107.60 ± 17.28 ms following the second stimulus (not shown). Comparing these sinks to their respective stimuli in the pair showed no significant difference in timing (*p* = 0.073, two-tailed paired *t*-test; Figure 6J) suggesting that each DG sink corresponds to one of the stimulus pulses. As with the SLM sinks, the significant difference in absolute latency relative to the first stimulus in the pair (Figure 6I) is attributable to the 30 ms ISI. Interestingly, unlike during SO, stimulation during theta states yielded only one prominent DG sink 22.00 ± 1.15 ms after the initial stimulus. A second DG sink may be occurring at a similar latency following the second stimulus, but it is apparently obscured by the prominent current source in DG that corresponds to the evoked SLM sink (Figure 6D).

We observed paired-pulse facilitation of the SLM sink only when stimulating during SO states (Figure 6K). Maximal SLM sink amplitude increased from −1.52 ± 0.41 to −3.58 ± 0.72 mV/mm^2^ (*p* = 0.029, two-tailed paired *t*-test) between pulses during SO (Figure 6K), while remaining unchanged during theta (sink 1: −2.57 ± 0.77; sink 2: −2.30 ± 0.77 mV/mm^2^; *p* = 0.69 two-tailed paired *t*-test; Figure 6L). This further suggests that fundamentally distinct circuits are being activated as a function of ongoing brain state: likely a direct input to HPC routing through the RE during SO states which shows facilitation, and an indirect cortico-entorhinal cortical route that ultimately activates the entorhinal cortices (EC) III inputs to SLM. DG sinks showed no facilitation either during SO or theta (SO: sink 1: −3.63 ± 0.90; sink 2: −3.80 ± 0.66 mV/mm^2^; *p* = 0.72; two-tailed paired *t*-test).

In order to test the idea of alternate pathways that did not involve the RE (both within and across states) we used chemogenetic means to inactivate the RE and assessed the subsequent influence on the IL-evoked potentials in HPC. Following systemic injections of CNO, and consistent with our prior work (Hauer et al., 2019), we observed an almost complete disappearance of both evoked current sinks at SLM during SO in DREADDs-expressing rats (post-CNO sink 1 amplitude: −0.12 ± 0.70 mV/mm^2^, *p* = 0.27, two-tailed paired *t*-test; post-CNO sink 2 amplitude: −0.084 ± 0.71 mV/mm^2^; *p* = 0.017 two-tailed paired *t*-test; Figures 6G,M). Although the depression of the first SLM sink was not significant, this was a likely consequence of the already low amplitude of this sink pre-CNO. In addition, we no longer observed any paired-pulse facilitation of the SLM sink post-CNO during SO (*p* = 0.62, two-tailed paired *t*-test). Across these analyses, the hSyn-mCherry expressing control rats showed no differences pre- vs. post-CNO administration (pre- vs. post-CNO SO sink 1: *p* = 0.82; pre- vs. post-CNO SO sink 2: *p* = 0.55; two-tailed paired *t*-tests). Taken all together, these data are consistent with the idea of a bisynaptic pathway from the mPFC to the HPC that is dependent on the integrity of the RE and that this pathway is functional during deactivated states.

In contrast to the disappearance of sinks at SLM, paired-pulse stimulation delivered during SO-states post-CNO continued to yield a prominent DG sink that was not significantly different from that evoked during control conditions pre-CNO in terms of either latency (pre-CNO: 84.00 ± 6.02 ms; post-CNO 87.33 ± 7.84 ms; *p* = 0.55, two-tailed paired *t*-test) or amplitude (pre-CNO: −2.52 ± 0.72 mV/mm^2^; post-CNO amplitude: −2.27 ± 0.92 mV/mm^2^; *p* = 0.39, two-tailed paired *t*-test; Figures 6G,O). We also continued to observe a second DG sink at a similar latency (113.33 ± 15.59 ms after the second stimulation) and with a similar amplitude (−2.90 ± 1.62 mV/mm^2^) to that recorded pre-CNO. This non-RE-dependent pathway likely involved an alternative route from mPFC, perhaps via the fronto-cortical circuits that is ultimately terminated in the layer II of EC which then profoundly innervates the DG via the perforant path.

Although marked alterations were observed at the level of SLM following the inactivation of the RE during SO, this was not the case during theta. Indeed, the overall pattern of evoked responses during theta appears remarkably unchanged when comparing cases pre- to post-CNO in DREADDs-expressing animals (Figure 6H). Neither the amplitudes nor latencies of the SLM sinks evoked during theta-states were significantly different post-CNO (post-CNO sink 1 amplitude: −4.22 ± 1.38 mV/mm^2^, *p* = 0.38, two-tailed paired *t*-test; post-CNO sink 2 amplitude: −3.46 ± 1.24 mV/mm^2^; *p* = 0.76, two-tailed paired *t*-test; Figures 6H,N; sink 1 latency: 58.33 ± 4.18 ms, *p* = 0.43, two-tailed paired *t*-test; sink 2 latency relative to stimulus 2: 54.33 ± 2.33 ms, *p* = 0.50, two-tailed paired *t*-test). As with the pre-CNO case, no paired-pulse facilitation was observed for the SLM sinks during theta (*p* = 0.53, two-tailed paired *t*-test). Furthermore, the initial DG sink observed pre-CNO was also unchanged (post-CNO amplitude: −1.02 ± 1.07 mV/mm^2^; *p* = 0.96, two-tailed paired *t*-test). The control rats (expressing the hSyn-mCherry vector) also did not show any differences pre- vs. post-CNO administration during theta (all *p*-values much >0.05). This suggests that IL stimulation during theta is entirely routed through a pathway that specifically does not involve the RE. Indeed, in partial contrast to the case with SO, this circuit may be biased to another fronto-cortical circuit that activates both layers II and III of the EC at different latencies.

Together, these data demonstrate that RE inactivation selectively impoverishes the mPFC-RE-SLM connection specifically during SO states. Activation of the mPFC during theta appears to target the HPC by an entirely alternate route.

### Hippocampal Excitation Is Modulated by the Ongoing Phase of Forebrain Rhythms

**Figure 7:**
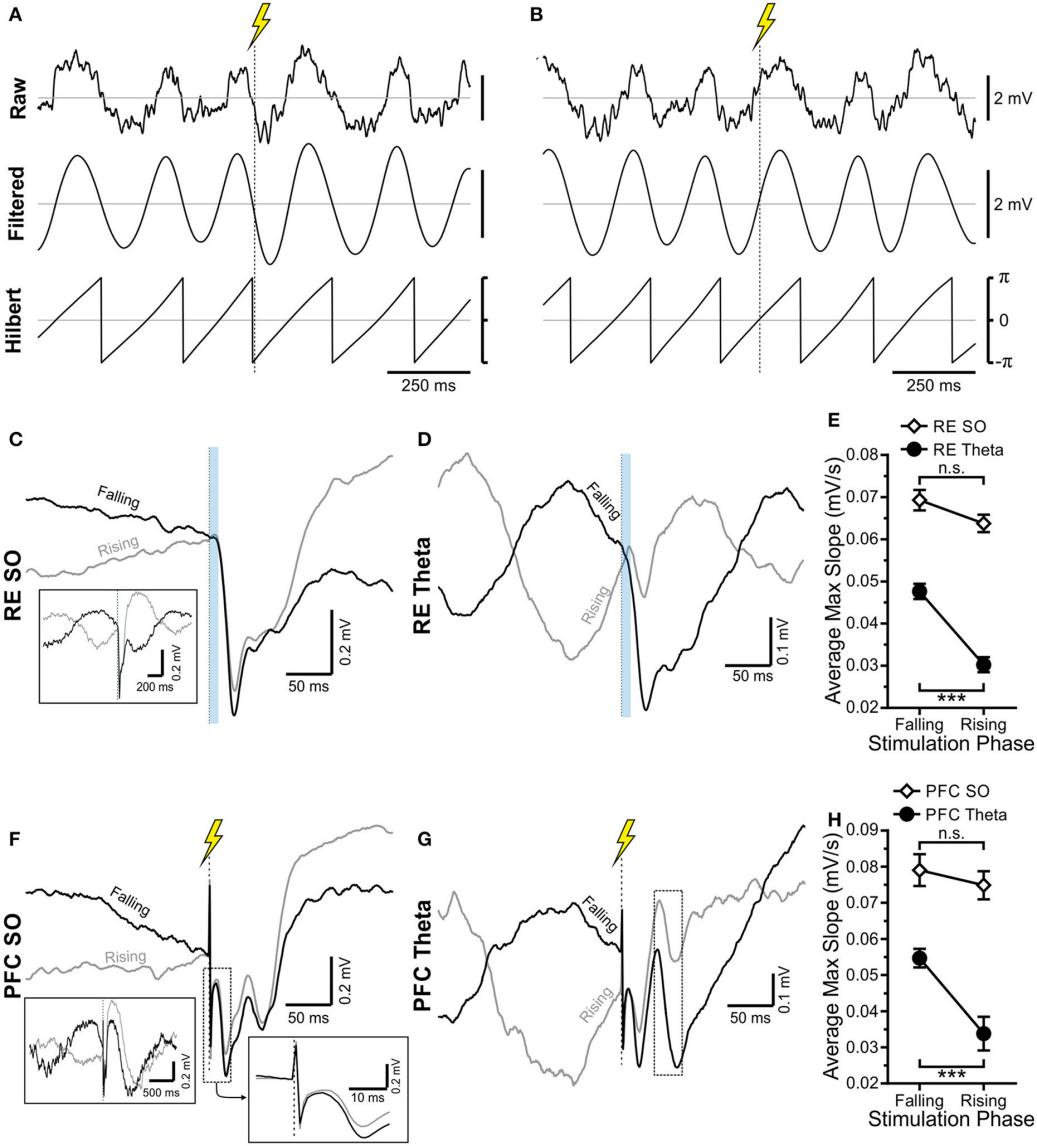
HPC is differentially excited by stimulation during the falling or rising phase of the theta and SO rhythms. **(A)** Raw local field potential recording, 3–6 Hz bandpass filtered signal, and Hilbert transformed signal of the same ~1.5 s of HPC theta. Stimulation is indicated by a vertical dashed line with a lightning bolt above it. Stimulation is being delivered during the falling phase of the oscillatory cycle. **(B)** Identical to **(A)** except that stimulation is being delivered on the rising phase of the oscillatory cycle. Scale bars are identical in **(A)**. **(C,D)** RE stimulation (10 ms optical pulse) being delivered during the falling (black) or rising (gray) phase of the ongoing SO cycle **(C)** and theta cycle **(D)**, with average, evoked local field potential (LFP) at SLM shown. Stimulation in both states produces an obvious evoked response, which appears larger when stimulation is delivered on the falling phase of the rhythm. **(C)** Provides a larger timescale for easier visualization of the rising or falling phase of the SO cycle. **(E)** Maximum evoked slope averaged over every IL stimulation trial during SO (unfilled black diamonds) and during theta (filled black circles). The maximum slope is significantly greater during theta when stimulation is delivered on the falling phase, while no significant difference is observed during SO. However, regardless of phase, stimulation during SO always yields a larger response. **(F,G)** The same as **(C,D)**, but with an electrical instead of the optical pulse, to infralimbic (IL) zones. Stimuli are delivered either on the falling (black) or rising (gray) phase of the SO **(F)** or theta **(G)** cycle. The dashed box in each corresponds to the approximate time window for evoked potential analysis. **(H)** As is the case with RE stim in **(E)**, IL stimulation delivered on the falling phase of theta (black, filled circles) produces a significantly larger maximum slope on average than equivalent stimulation delivered during the rising phase. No significant difference between stimulation phases is observed during SO (hollow black diamonds), although stimulation during SO always yields a larger response than stimulation during theta, regardless of the oscillatory phase.

By tracking the amplitude of field potential responses evoked during either RE or IL stimulation across random phases of the ongoing SO or theta rhythm, we also noted a phase dependency of the HPC response at SLM (Figure 7). This was first shown by separating the stimuli occurring during either the falling or rising phase of the oscillation of interest (Figures 7A,B). As shown for the example in Figure 7D, the magnitude and slope of the evoked potential was greater when RE stimulation was delivered during the falling, as compared with the rising phase of the theta rhythm (falling: 0.048 ± 0.0018 mV/s; rising: 0.030 ± 0.0018 mV/s; *p* < 0.0001, two-tailed *t*-test assuming equal variances; Figures 7D,E). This was the also the case for stimulation of IL during theta (Figure 7G) (falling: 0.055 ± 0.0026 mV/s; rising: 0.034 ± 0.0047 mV/s; *p* < 0.001, two-tailed *t*-test assuming equal variances; Figures 7G,H). Interestingly, the magnitude of the difference between the falling phase as compared with the rising phase during theta was similar whether stimuli were delivered to RE (0.017 mV/s) or IL (0.021 mV/s).

Although the amplitude and slope of evoked potentials recorded across different phases of the SO showed a similar direction, with the falling phase being larger than the rising phase (Figures 7C,F), these values on average were not significantly different with stimulation of either the RE (falling: 0.069 ± 0.0024 mV/s; rising: 0.064 ± 0.0021 mV/s; *p* = 0.083, two-tailed paired *t*-test; Figures 7C,E) or IL (falling: 0.079 ± 0.0044 mV/s; rising: 0.075 ± 0.0039 mV/s; *p* = 0.55, two-tailed paired *t*-test; Figures 7F,H). However, in all cases (rising or falling phase, with stimulation at either RE or IL), stimulation during SO yielded a much larger response than the equivalent stimulation during theta (Figures 7E,H).

**Figure 8:**
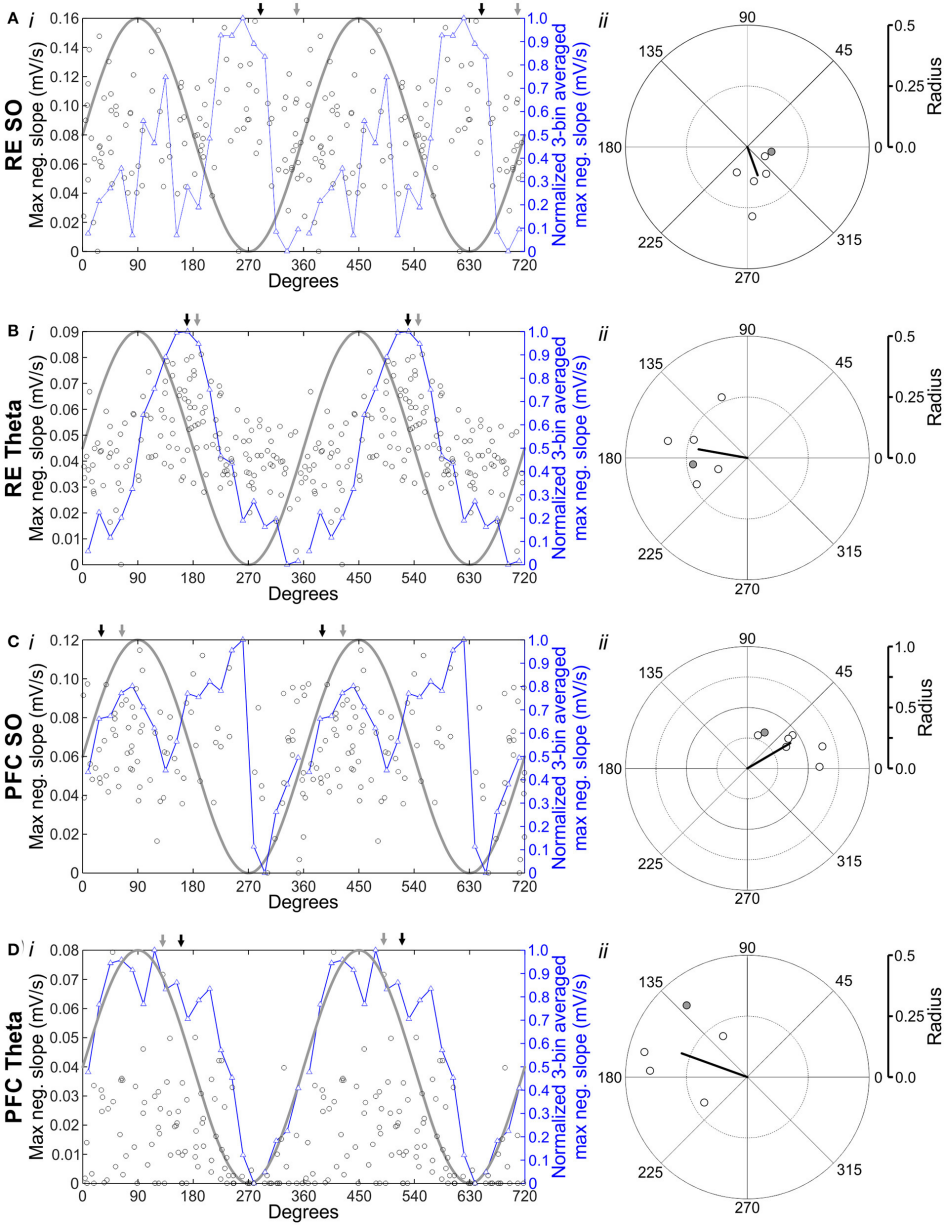
Hippocampal excitation is modulated by the phase of the ongoing forebrain rhythm. **(Ai)** Stimulating RE during SO evokes an HPC response with a variable maximum slope. Each black hollow circle represents the maximum negative slope evoked by stimulation of RE, which fluctuates as a function of the phase in the oscillatory cycle that the pulse was delivered at. Phase values were organized into 20 equivalent 18° bins, and the average maximum slope for each bin was calculated. The normalized 3-bin average (a rolling average of the center bin and two flanking bins) was computed and plotted (hollow blue triangles and blue line). An idealized sine wave is overlaid (gray line) to aid in visualizing phase preference, as indicated by the greater or lesser maximum evoked slope. All data (including the 3-bin average and the sine wave) are repeated a second time to illustrate two full cycles. The Gray arrow indicates mean phase preference for this experiment; the black arrow indicates mean phase preference across all experiments. **(Aii)** Circle plot of preferred phase of HPC response (as measured by maximum evoked slope) to RE stimulation during SO. The thick, black line indicates the mean angle (°) and the strength of phase preference computed via circular Rayleigh statistics. Gray, filled circle corresponds to the data depicted in **(Ai)**. **(B)** Identical to **(A)**, except that stimulation was delivered optogenetically to RE during theta. **(C)** Identical to **(A)**, except that stimulation was delivered electrically to IL, during SO. **(D)** Identical to **(C)**, except that electrical IL stimulation was delivered during theta.

We expanded upon this analysis to determine on a stimulus-by-stimulus basis what particular phase might yield the greatest response across both stimulus locations and brain states (Figure 8). To visualize the average phase preference, we further subdivided the occurrence of stimuli into 20 equally sized bins (18° wide) across the entire oscillatory cycle. To assist in smoothing the bin-to-bin comparisons (which could appear artificially noisy in the case of relatively few stimulations in a given bin), we computed the normalized average of three bins (the bin of interest, plus the bins on either side) and compared that with an idealized superimposed sine wave to represent the oscillatory cycle of ongoing rhythmic field activity (Figure 8, left column, normalized 3-bin average in blue, sine wave in gray).

As shown in Figure 8, we observed a prominent and significant degree of phase coupling of the maximum evoked slope to the ongoing field potential oscillation across stimulation sites and brain states. Stimulating RE during SO (Figure 8A) showed a preference for responding during the positive rising phase of the field oscillation (in this example, angle: 348.68°; radius: 0.10; *n* = 105 stimuli; Rayleigh *p* = 0.58; Figure 8Ai). The window for the maximal slope of the evoked potential corresponding to the first prominent negative deflection at SLM (from 11.17 ± 1.64 to 25.50 ± 3.08 ms). Preference for the rising phase of the SO cycle was also observed on average (individually significant in 1 of 6 experiments; overall average preferred angle: 70.10°; overall average radius: 0.12; *N* = 6 experiments; Rayleigh *p* = 0.014; Figure 8Aii). Conversely, the stimulation of RE during theta (Figure 8B) displayed a somewhat stronger phase preference for earlier in the field cycle, during the falling phase. This held true both in this example (angle: 186.73°; radius: 0.22; *n* = 150 stimuli; Rayleigh *p* = 0.013; Figure 8Bi), and on average (individually significant in 4 of 6 experiments; overall average preferred angle: 169.95°; overall average radius: 0.20; *N* = 6 experiments; Rayleigh *p* = 0.016; Figure 8Bii). The window chosen for maximal slope similarly corresponded to the initial negativity at SLM (from 12.17 ± 2.40 to 23.00 ± 2.25 ms).

During SO, the stimulation of IL (Figure 8C) showed a prominent preference for responding during the positive rising phase of the cycle (in this example, average preferred angle: 64.19°; radius: 0.33; the number of stimuli: 86; Rayleigh *p* = 0.0061; Figure 8Ci). This was consistent across 7 of 7 rats (overall average preferred angle: 30.46°; overall average radius: 0.41; *N* = 7 experiments; Rayleigh *p* = 0.00014; Figure 8Cii). Here again, the initial SLM negativity was chosen for slope analysis (from 11.43 ± 0.57 to 25.71 ± 3.08 ms). Phase preference was less obvious across ongoing theta, but still showed a pattern of maximal excitation similar to that observed during stimulation of RE. Stimulating IL during theta (Figure 8D) yielded a maximal response during the falling phase of the cycle (in this example, angle: 130.22°; radius: 0.39; *n* = 121 stimuli; Rayleigh *p* = 0.047; Figure 8Di). This non-statistically significant pattern held true on average as well (individually significant in 1 of 5 experiments; overall average preferred angle: 160.15°; overall average radius: 0.29; *N* = 5 experiments; Rayleigh *p* = 0.062; Figure 8Dii). Importantly, given the delayed maximal response at SLM following IL stim during theta specifically, the window for evoked potential analysis was later than for SO or RE stimulation (from 27.00 ± 1.18 ms to 39.40 ± 0.60 ms).

## Discussion

Understanding the neural dialogue between extended brain regions that have parallel functional relationships for cognition is important for understanding integrative brain operation. Of particular interest for episodic memory are the connections between the mPFC and the HPC. Although much is known about the anatomy of their interactions, much less is known about their functional relationships during ongoing and shifting exigencies. In the present work, we have demonstrated that global forebrain state fundamentally alters the way in which the mPFC and HPC interact. Furthermore, we have demonstrated that this state-dependent interaction is critically modulated by an interposed structure, the thalamic RE. Our study underscores the importance of delineating the ways in which brain circuitry might be modulated by shifts in overall state.

### Prefrontal and Thalamic Excitation of the HPC

Stimulating RE during baseline conditions reliably produced a hippocampal current sink that was maximal at SLM. This excitation of CA1 distal dendrites is consistent with previous reports (Dolleman-Van der Weel et al., 1997; Dolleman-van der Weel et al., 2017; Bertram and Zhang, 1999; Morales et al., 2007; Hauer et al., 2019; Vu et al., 2020). However, what is novel here is the demonstration of enhanced excitability during SO as compared with theta states. This was confirmed by the stimulation of the RE-to-HPC afferent fibers in the CB, revealing the same pattern of enhanced excitation during SO. RE stimulation also produced a somewhat later sink in DG across both states, a finding that again is consistent with past work (Hauer et al., 2019; Vu et al., 2020). While a direct route from RE to DG has not been demonstrated anatomically (Herkenham, 1978; Wouterlood et al., 1990) we suspect that this late DG sink arises from a likely disynaptic circuit involving RE projections to lateral and medial EC, and subsequently to DG *via* the perforant path (Yanagihara et al., 1987; Wouterlood, 1991). Indeed, and in line with preliminary studies in our own laboratory (Hauer, Pagliardini, and Dickson, unpublished observations), the stimulation of the ventral midline thalamus in urethane-anesthetized rats has been previously shown to evoke a significant monosynaptic excitatory potential in EC (Zhang and Bertram, 2002). Specifically, ventral midline thalamic stimulation produced the largest amplitude responses in more lateral areas of EC layer II and III (Zhang and Bertram, 2002). Other work describing a significantly temporally delayed DG sink following RE stim also attributed it to layer II activation of both lateral and medial EC (Vu et al., 2020). This is further supported by anatomical work demonstrating that RE fibers innervate the dendrites of principal cells in both layers II and III of EC (Wouterlood, 1991). As such, an alternate (shorter and faster) ventral thalamo-temporal pathway presumably exists for RE-EC afferents to travel, creating a disynaptic RE-EC-DG circuit.

Stimulating IL similarly yielded a prominent sink at SLM during both SO and theta, although at a significant delay during the latter. We also observed a delayed DG sink during SO, but a temporally earlier sink during theta. Chemogenetic inactivation of RE only impacted responding during SO, robustly diminishing the SLM sink while leaving the DG sink unperturbed. The silencing of RE had no impact on HPC responses during theta, suggesting that an alternate circuitry is engaged for PFC-HPC dialogue during activated states. Past work has shown that ventromedial PFC stimulation modulates HPC LFPs and the coherence between PFC and HPC (Jia et al., 2019). Both IL and PL also densely innervate lateral and medial EC almost equally, particularly at the pyramidal cells of layer Vb (Witter et al., 2017). From here, information is outputted from layer Va to CA1/subiculum and beyond for further processing, while a hippocampally processed copy of the original input information is relayed back to layer Vb. Layer Vb as such may be well-positioned to integrate these inputs with additional sets of information and send these representations to layers II and III (Witter et al., 2017). Layers II and III are also major modulators of HPC activity via the perforant pathway (layer II → DG) and temporoammonic pathway (layer III → SLM). Further study is required to precisely characterize the state-dependencies of EC cell lamina, both in terms of the layer II and III projections to HPC, but also in terms of the potential role of layer Vb in modulating and integrating these inputs. Indeed, past work has demonstrated differential responsiveness to slow rhythmic stimulation compared with theta-burst stimulation in deep vs. superficial layers of EC (Yun et al., 2002). It is likely that the DG sink that remains even after RE is chemogenetically inactivated is a consequence of these dynamic inputs, suggesting that the RE-SLM pathway may be the preferred access point to the HPC for cortico-thalamic information during SO.

### Importance of State

It is well-known that the intrinsic responsiveness of single neurons is subject to diverse neuromodulatory influences that are correlated themselves to brain state changes (Kaczmarek and Levitan, 1987; Steriade, 2001; Marder et al., 2014). It is also well-known that local networks can modify their collective properties based on alterations in neuromodulatory influences again related to brain state changes (Steriade, 2001; Marder, 2012). Our present work documents how inter-areal forebrain circuit interactions are changed in fundamentally different ways based upon state. As such, our work further highlights the importance of both monitoring and reporting on brain state as a function of understanding brain operation since this is both a critical theoretical and experimental concern. In our present study, not only have we documented striking changes in terms of neural responding in the mPFC to HPC circuit that depend on state, but we have also shown that ongoing cycles of circuit rhythms expressed within these states are another significant aspect of altered responsiveness.

### State-Dependent Modulation of mPFC-HPC Circuitry

The interaction of the mPFC and HPC is crucial for episodic mnemonic functions (Preston and Eichenbaum, 2013). It is important to note that these memory-related functions are not only engaged during wakefulness when behavioral performance is required and learning can occur but also during the subsequent offline periods that follow learning, such as sleep (Eichenbaum, 2004; Stickgold, 2005; Dickson, 2010). The distinct and dynamic electrographic patterns of activity that occur during sleep, especially within and between the cycles of REM and non-REM periods, are likely candidates for examining the interactions between mPFC and HPC that might facilitate long-term storage and consolidation of declarative memories (Marshall et al., 2006; Klinzing et al., 2019).

Urethane anesthesia is an ideal model for studying brain-wide interactions that occur during sleep given how closely it resembles the natural central patterns of unconsciousness (Ward-Flanagan and Dickson, 2019). To date, only urethane allows for spontaneous and dynamic changes in the brain state that parallel the alternations between REM and non-REM sleep. Not only are individual electrographic states similar to each of these respective stages of sleep, but also the timing of alternations and other corresponding peripheral physiological changes mimic those of natural sleep (Clement et al., 2008; Whitten et al., 2009; Pagliardini et al., 2012). Indeed, these same alternations are also observed in urethane-anesthetized mice (Pagliardini et al., 2013b). The urethane model is therefore ideally suited to study the central and peripheral dynamics of natural sleep, particularly with respect to the spontaneous alternation between distinct activity states. This considered, however, future work should seek to replicate the findings presented here in drug-free, naturally sleeping animals to ensure that the same state-dependent modulations occur during naturalistic sleep/wake cycles.

Our findings here demonstrate that the spontaneously expressed and state-dependent patterns of activity across the forebrain appear to differentially engage memory-relevant circuitry and regulate hippocampal excitability. Despite apparent tonic excitation of RE cells during theta (Morales et al., 2007; Hauer et al., 2019), optogenetic activation of RE produced a smaller SLM response as compared with SO. Perhaps, more surprising was that the pattern of responding in this same region following mPFC stimulation was entirely different across states and suggestive of an alternate re-routing of inputs. The stimulation of the mPFC during SO produced a large-amplitude SLM-located excitation at short latencies that displayed clear paired-pulse facilitation. This response was eliminated following chemogenetic RE inactivation. In contrast, during theta states, we observed a significantly longer latency excitation, which failed to show paired-pulse facilitation. This latter response remained intact following RE inactivation. Our results suggest that the more direct influence of the mPFC input to the HPC via the RE was greatly impoverished during theta states and that mPFC-HPC interactions were mediated instead through a cortico-entorhinal circuit. Altogether, this indicates that the RE is ideally situated to modulate the flow of information from the mPFC to the HPC across deactivated and activated states such that during slow oscillatory states, responding through this bi-synaptic circuit is optimized, whereas, during theta, this circuit is bypassed in favor of a cortico-entorhinal circuit. It is worth emphasizing that the role of the RE in mediating this long-range information transfer during theta is still important, as its intrinsic activity appears to select for a different circuitry to be engaged which may be vital for waking behavioral or mnemonic processes.

We have previously shown that during SO states, the RE exhibits slow, rhythmic single-unit firing that is coupled to the ongoing mPFC SO (Hauer et al., 2019). Conversely, we showed that during theta states, RE neurons fire tonically and arhythmically [also shown by Morales et al. (2007)]. The differential activity and responding of the RE to mPFC inputs during theta could be related to the depolarizing influence of increased cholinergic neuromodulation during activated states (Hasselmo, 2006; Clement et al., 2008). With a profound tonic activation of RE neurons, it may well be the case that the influence of mPFC inputs are effectively filtered out. Cholinergic neuromodulation is also able to pre-synaptically depress excitatory transmission (Picciotto et al., 2012; Colangelo et al., 2019) which might work at both mPFC terminals in RE and/or RE terminals in HPC.

The relevance of advantaged RE-mediated inputs to the HPC during slow-wave states likely plays an important role in global SO synchronization. Indeed, based on the strong coupling of RE unit activity to mPFC slow waves, and the loss of synchronization of mPFC and hippocampal SO activities with RE inactivation (Hauer et al., 2019), it would appear that the RE is an important conduit for mPFC information to arrive at the HPC during slow-wave states. With respect to SO-related coupling throughout the forebrain, the mPFC has been framed as a key player. It has been suggested to play a pacemaking role in forebrain SO coordination since it often appears as the source of propagating SO waves across the cortex that can additionally be entrained by electric or magnetic field stimulation delivered to frontal regions (Massimini et al., 2004, 2007; Marshall et al., 2006; Greenberg and Dickson, 2013; Greenberg et al., 2016). Here we suggest that this potential mPFC pacemaking role can be extended to include the coordination of the hippocampus, via the interposed RE.

### Oscillatory Phase Preference as a Mechanism for Memory Formation

The ongoing state was an important modulator of HPC excitability and the pattern of responding, but so too was the phase of the oscillatory field cycle whether during SO or theta. This type of phase preference, or active modulation of responding as a function of the rhythmic cycle, provides the circuitry involved with an even more fine-grained temporal mechanism for influencing information processing (Jacobs et al., 2007; Sauseng and Klimesch, 2008; Schall et al., 2008; Cox et al., 2020). Stimulation of either IL or RE yielded a larger HPC evoked potential slope during the falling phase of the theta cycle compared with the rising phase. In both theta and SO, excitability was systematically modulated across the phase of the cycle. Regardless of phase, stimulation during SO produced a larger response than did stimulation during theta. Modulated CA1 responding by phase has been shown before during theta (Wyble et al., 2000), as well as during SO (Schall et al., 2008). However, we are the first to demonstrate that a cyclical modulation of excitability in the mPFC-RE-HPC circuitry specifically.

The rising and falling phases of the extracellular field potential rhythm are a consequence of net current flow entering or leaving the extracellular medium being recorded from, creating windows of enhanced synaptic efficacy (Buzsaki et al., 2012). Our results show that this type of rhythmic modulation is occurring across both states within the broad mPFC-RE-HPC circuitry. There is an optimal phase window during which stimulation of IL may most effectively relay information to HPC, presumably *via* the RE during SO, and via EC during theta. This is consistent with our schema of the RE as a key relay node for (in particular) slow oscillatory information, where the precise, phase-dependent timing of inputs from mPFC to RE, and from RE to HPC is reliant on the SO-coupled unit activity of RE (Hauer et al., 2019). Moreover, we suggest here that the forebrain state may differentially bias this circuitry during theta, engaging an alternative entorhinal pathway.

It is difficult to surmise the full implications of this state- and phase-dependent modulation in the mPFC-RE-HPC circuitry, although certainly, the relative timing of discharging neurons in this circuit would have marked functional implications for synaptic plasticity and memory formation as a whole (Hyman et al., 2003; Dolleman-van der Weel et al., 2017; Vu et al., 2020).

### Functional Relevance of State-Dependent Modulation of mPFC-HPC Inputs

Conventional models of frontal-hippocampal interplay in episodic memory describe the HPC as forming and replaying episodes, while the mPFC engages contextual representations that link related memories, in order to retrieve memories appropriate for any given context (Preston and Eichenbaum, 2013). One likely mechanism by which these disparate sites could communicate most effectively is by coordinated SO activity (Born, 2010). Given the prominence of the SO during the deep stages of slow-wave sleep, and how CA1 excitability to both RE and mPFC stimulation is maximal during this form of activity, we suggest that the circuitry corresponding to both the input and output of this hippocampal region is preferentially biased during SO states as compared with during theta (Schall et al., 2008). As we have previously reported, spontaneous slow-wave activity between frontal neocortical regions and the HPC is dynamically coordinated during SO states (Wolansky et al., 2006), which supports the idea that neural interactions between these disparate structures could be systematically synchronized or de-synchronized on a cycle by cycle basis. This coupling (and, indeed, de-coupling) of cortical and hippocampal ensembles during SO is an ideal platform for the associative and activity-dependent processes of both long-term potentiation (LTP) and long-term depression (LTD), respectively (Dickson, 2010). In this way, neural activity during the SO can provide a dynamic window for the process of declarative memory consolidation to occur in a spike-timing-dependent manner (Dan and Poo, 2004).

In this context, the SO itself has been recognized as an important element in sleep-dependent memory consolidation (Born, 2010; Diekelmann and Born, 2010). It is thought that it temporally organizes both local (intra-areal) and global (inter-areal) replay of newly-formed neural ensembles (Diekelmann and Born, 2010; Staresina et al., 2015; Miyamoto et al., 2017; Klinzing et al., 2019). Indeed, replay is most intensive during slow-wave states (Pavlides and Winson, 1989; Ji and Wilson, 2007; Born, 2010). As alluded to above, in this way, the SO-dependent reactivation of neural sequences corresponding to recently-experienced episodic memory representations could be synchronized in a fashion that would lead to further activity-dependent synaptic facilitation. This type of “neural rehearsal” would further strengthen connections between ensemble elements to promote “to-be-remembered” episodic representations. Consistently, we also demonstrated that the frontal cortex shows an enhanced response to RE stimulation during the SO as well. The phase-dependent coordinated replay of related cellular ensembles in both the mPFC and the HPC could serve as a reverberative strategy to enhance the activity-dependent coupling of these neural representations. Having the return loop back to the mPFC from the HPC (Jay et al., 1989; Hoover and Vertes, 2007) involved as well-might create a self-sustaining and repetitive reverberation that would be well-positioned to either strengthen or weaken a set of neuronal assemblies in both the mPFC and HPC, depending upon the relative phasing of activations. Although untested in our study, it would be of significant interest to understand how hippocampal output, either via CA1 or subiculum, is modulated by brain state at the level of the mPFC.

Regardless of how memory representations might be solidified across slow-wave states, the synchronized coupling of SO activity between the prefrontal cortical regions and the HPC is primarily dependent upon the interposed activity in the RE, as we have previously shown (Hauer et al., 2019). In this way, the RE is vitally positioned to either synchronize or desynchronize cellular assemblies across the mPFC and HPC during slow-wave states. Indeed, it would also appear that phasic information from the RE is rhythmically biased in this circuit with maximal excitability at very nearly the exact phase of the SO (~270°) at which RE units show their maximal discharge preference (Hauer et al., 2019). This means that RE input is optimized for maximal hippocampal effect during the SO and it is likely that this is what allows it to coordinate and synchronize hippocampal slow oscillatory activity during slow-wave states.

We feel that the functional relevance of the theta state in the mPFC-RE-HPC circuitry may be related to the increased influence of cholinergic modulation present. Brain-wide release of acetylcholine has been suggested as a signal to update existing memories with new relevant information, or to encode new memories entirely (Hasselmo et al., 1996; Hasselmo, 2006). In this way, the RE could act as a switch, biasing information to be relayed through a cortico-entorhinal route during high acetylcholine, theta rhythmic “encoding” states for new memories or for updating existing ones. This is consistent with the RE being an integral mediator of the prefronto-hippocampal theta synchrony during behavioral tasks, such as spatial working memory paradigms (Hallock et al., 2016; Maisson et al., 2018). Conversely, during SO, the lower levels of acetylcholine in the extracellular *milieu*, combined with the slow rhythmic firing of RE units that are coupled to the ongoing mPFC SO (Hauer et al., 2019) promotes a “consolidating” state, updating representations in the HPC with new rules and binding related episodes together for easier retrieval. The way RE and HPC interact is completely different between states, not only because of the activity of RE itself but because the entire prefronto-thalamo-hippocampal circuitry is changing as well.

## Conclusion

Here, we show that the PFC-HPC communication is fundamentally different between states, and that the anatomically interposed thalamic RE has a critical role in mediating this disparity. Distinct circuits can be engaged as a function of this ongoing forebrain state, with slow oscillatory states likely being ideal platforms for forebrain information exchange. To this end, the RE reliably produces a larger response in the CA1 during SO activity and is critical in the standard PFC-to-HPC circuitry. During activated theta states, a cortico-cortical circuit *via* the EC may instead be the preferred pathway of information transfer. However, both states show a rhythmical modulation of excitability dependent on the oscillatory phase of the ongoing rhythm. Together, our data demonstrate that the RE has a critical role in mediating PFC-HPC information transfer during slow oscillatory states and that the neural circuitry engaged during theta states is fundamentally different. This has marked implications for the circuitry involved in memory formation, and how the RE may be differentially engaged across deactivated and activated states to underlie it.

## Data Availability Statement

The raw data supporting the conclusions of this article will be made available by the authors, without undue reservation.

## Ethics Statement

Animal studies were reviewed and approved by the Biological Sciences and/or Health Sciences Animal Policy and Welfare Committees (AUP 092 and AUP 461) of the University of Alberta.

## Author Contributions

BH, SP, and CD designed research. BH performed research. BH and CD analyzed data, wrote, and edited the paper. SP edited the paper. All authors contributed to the article and approved the submitted version.

## Funding

This work was supported by Natural Sciences and Engineering Research Council of Canada (NSERC) Discovery grants 22016-06576 and 2021-02926 to CD and NSERC grant 435843 to SP. BH was supported by an NSERC Doctoral Postgraduate Scholarship.

## Conflict of Interest

The authors declare that the research was conducted in the absence of any commercial or financial relationships that could be construed as a potential conflict of interest.

## Publisher's Note

All claims expressed in this article are solely those of the authors and do not necessarily represent those of their affiliated organizations, or those of the publisher, the editors and the reviewers. Any product that may be evaluated in this article, or claim that may be made by its manufacturer, is not guaranteed or endorsed by the publisher.
